# *De novo* transcriptome sequencing and comprehensive analysis of the drought-responsive genes in the desert plant *Cynanchum komarovii*

**DOI:** 10.1186/s12864-015-1873-x

**Published:** 2015-10-06

**Authors:** Xiaowen Ma, Ping Wang, Sihong Zhou, Yun Sun, Nana Liu, Xiaoning Li, Yuxia Hou

**Affiliations:** College of Science, China Agricultural University, No.2 Yuanmingyuan West Road, Beijing, 100193 China

**Keywords:** *Cynanchum komarovii*, Drought-stress response, Illumina sequencing, Transcriptome

## Abstract

**Background:**

*Cynanchum komarovii* Al Iljinski is a xerophytic plant species widely distributing in the severely adverse environment of the deserts in northwest China. At present, the detailed transcriptomic and genomic data for *C. komarovii* are still insufficient in public databases.

**Results:**

To investigate changes of drought-responsive genes and explore the mechanisms of drought tolerance in *C. komarovii*, approximately 27.5 GB sequencing data were obtained using Illumina sequencing technology. After *de novo* assembly 148,715 unigenes were generated with an average length of 604 bp. Among these unigenes, 85,106 were annotated with gene descriptions, conserved domains, gene ontology terms, and metabolic pathways. The results showed that a great number of unigenes were significantly affected by drought stress. We identified 3134 unigenes as reliable differentially expressed genes (DEGs). During drought stress, the regulatory genes were involved in signaling transduction pathways and in controlling the expression of functional genes. Moreover, *C. komarovii* activated many functional genes that directly protected against stress and improved tolerance to adapt drought condition. Importantly, the DEGs were involved in biosynthesis, export, and regulation of plant cuticle, suggesting that plant cuticle may play a vital role in response to drought stress and the accumulation of cuticle may allow *C. komarovii* to improve the tolerance to drought stress.

**Conclusion:**

This is the first large-scale reference sequence data of *C. komarovii*, which enlarge the genomic resources of this species. Our comprehensive transcriptome analysis will provide a valuable resource for further investigation into the molecular adaptation of desert plants under drought condition and facilitate the exploration of drought-tolerant candidate genes.

**Electronic supplementary material:**

The online version of this article (doi:10.1186/s12864-015-1873-x) contains supplementary material, which is available to authorized users.

## Background

Drought is one of the most severe and wide-ranging environmental abiotic stresses that significantly threats to agriculture and influences germination, growth, development of plants [1]. In many plant species, seed germination and subsequent seedling growth are the stages most sensitive to environmental stresses [2]. Furthermore, seedling establishment could be particularly affected by the drought stress. The response to drought stress is a sophisticated process in plant, which involves thousands of protein-coding genes and biochemical-molecular mechanisms [3, 4]. Exposed to drought stress, plants appear to activate a diverse set of physiological, metabolic and defense systems by altering genes expression patterns for responding and adapting to stress. Drought resistance in plants is based on the expression of several stress-inducible genes, which are generally classified into two groups: those encoding function proteins directly protect plants against environmental stresses; and those encoding regulatory proteins [5, 6]. Many studies on the responses to drought from genes to the whole plant have been performed [3]. However, the knowledge about drought response in desert plants was only studied in some species [7–9].

*Cynanchum komarovii* Al Iljinski is a perennial, erect, caespitose herbaceous plant belonging to the family of *Asclepiadaceae*, mainly distributes in the semi-fixed sand dunes of secondary desert and in the quicksand areas of harsh deserts of Inner Mongolia and Ningxia Autonomous Regions, as well as Gansu Province. *C. komarovii*, being the indicator species of the last stage of grassland retrogressive succession, adapts to the dry and barren environment in the desertification process and plays a vital role in maintaining desert ecosystems [10]. As a wild desert plant, *C. komarovii* possesses strong resistance to abiotic stresses, including drought, UV, high light and high temperature, which makes it a valuable non-model species to investigate the stress tolerance mechanisms and to discover the stress-tolerance candidate genes [11]. According to previous studies, seedlings of *C. komarovii* treated with high concentrations of polyethylene glycol-6000 (PEG6000), showed a relative water content (RWC) decrease and malondialdehyde (MDA) increase, and showed the increases in activities of superoxide dismutase (SOD), peroxidase (POD), and catalase (CAT) [12]. Meanwhile, the contents of betaine, starch, proline, and soluble sugar (i.e., fructose, sucrose, trehalose) increased obviously, suggesting that the accumulation of some osmoprotectant may play an important role in osmotic adjustment during drought treatment and the introduction of osmoprotectant synthesis pathways may be a potential strategy for improving the stress tolerance of desert plant [13–15]. Therefore, it would be beneficial and imperative to perform studies whether the xerophytes have unique adaptive strategies in the morphological, physiological and molecular traits. However, the knowledge about the molecular mechanism of drought tolerance in *C. komarovii* is still poorly understood, due to lack of detailed genetic and sequence information for this non-model plant.

During the last few years, the next generation sequencing (NGS) technology has provided opportunities for the efficient, economical, and comprehensive analysis of plant with and without a reference genome in greater depth than ever before [16]. This powerful high-throughput sequencing technology has been widely applied to transcriptome sequencing, which is an approach to generate functional genomic data, and to discover DEGs in different cultivars, organs, or different treatment conditions [17–19]. To better understand the drought tolerance molecular mechanisms of *C. komarovii*, we performed a transcriptome sequencing using Illumina HiSeq^™^ 2000 sequencing platform and compared the transcriptome of drought-treated and control plants to explore the global transcriptional changes in root tissues, and to identify candidate genes involved in drought tolerance. This is the first large-scale reference sequence data of *C. komarovii*, which enlarge the genomic resources of this species and will facilitate the further novel genes discovery research in *C. komarovii*.

## Results and discussion

### Illumina sequencing and reads assembly

**Fig. 1:**
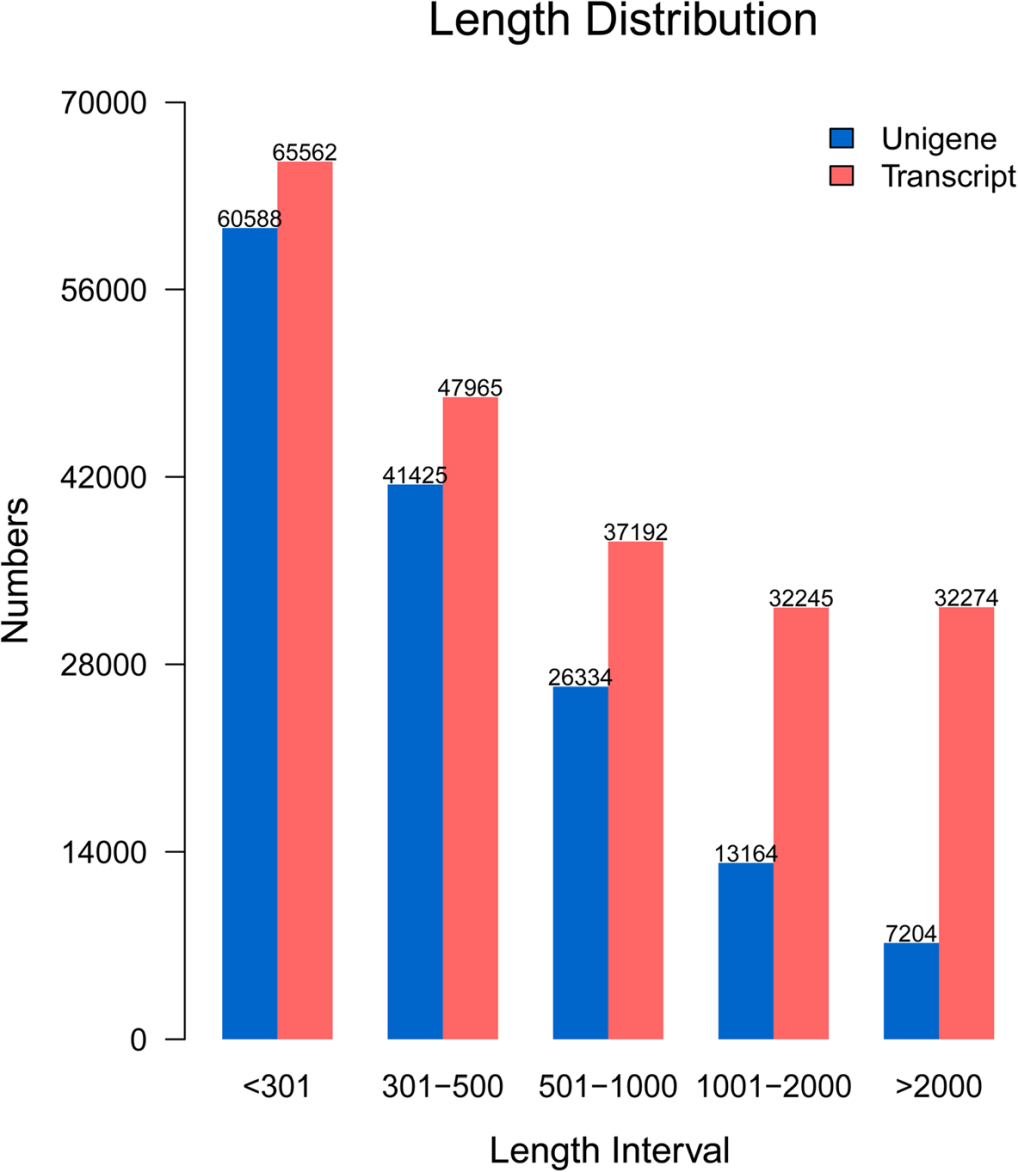
The length distribution of the transcripts and unigenes. The length distribution of the transcripts (red), and unigenes (blue)

To investigate the transcriptomic responses to drought-stress in *C. komarovii*, four cDNA libraries were generated from mRNA of drought-treated (DT) and control (CK) samples, which were sequenced using Illumina deep-sequencing HiSeq^™^ 2000. All reads were deposited in the National Center for Biotechnology Information (NCBI) and can be accessed in the Short Read Archive (SRA) database under accession number SRP057292. Among all the raw reads, 97 % had Phred-like quality scores at the Q20 level (an error probability of 1 %). After removing adapters, low-quality sequences and ambiguous reads, we obtained approximately 70 million, 68 million, 66 million and 70 million clean reads from the drought-treated samples (DT_1 and DT_2), and control samples (CK_1 and CK_2), respectively (Table 1). Totally, 275,425,782 clean reads (including CK and DT samples) were used to assemble the transcriptome data using the Trinity method. Using overlapping information in high-quality reads, 215,238 transcripts and 148,715 assembled unigenes were generated (Table 2). Over 83 % reads could be mapped back to the assembled transcripts, indicating a high quality of assembly. All unigenes were longer than 200 bp and 102,013 of them were 200 to 500 bp. Also, 20,368 of unigenes were longer than 1000 bp. The length distribution of the transcripts and unigenes is shown in Fig. 1.

**Table 1:** Summary of sequences analysis

Sample	Raw reads	Clean reads	Clean bases	Error (%)	Q20 (%)	Q30 (%)	GC Content (%)
CK_1_1	34,754,202	33,279,869	3.33G	0.03	97.38	91.84	43.12
CK_1_2	34,754,202	33,279,869	3.33G	0.04	96.52	90.36	43.16
CK_2_1	37,597,921	35,233,977	3.52G	0.03	97.44	91.95	42.81
CK_2_2	37,597,921	35,233,977	3.52G	0.03	96.74	90.81	42.85
DT_1_1	36,559,636	35,218,245	3.52G	0.03	97.37	91.84	42.67
DT_1_2	36,559,636	35,218,245	3.52G	0.04	96.49	90.32	42.69
DT_2_1	35,239,113	33,980,800	3.40G	0.03	97.43	92.00	42.65
DT_2_2	35,239,113	33,980,800	3.40G	0.04	96.54	90.47	42.67
Total	288,301,744	275,425,782	27.54G				

CK_1 and CK_2: Controlled sample (two independent biological replicates)

DT_1 and DT_2: Drought treated sample (two independent biological replicates)

CK_1_1: Reads sequencing of controlled sample 1 from the left

CK_1_2: Reads sequencing of controlled sample 1 from the right

Q20: The percentage of bases with a Phred value >20

Q30: The percentage of bases with a Phred value >30

**Table 2:** Length distribution of assembled transcripts and unigenes

	Transcripts	Unigene
Min Length(nt)	201	201
Mean Length(nt)	1,004	604
Median Length(nt)	462	344
Max Length(nt)	17,471	17,471
N50	2,021	862
N90	351	255
Total Number	215,238	148,715
Total Nucleotides	216,140,823	89,843,452

### Functional annotation and classification of the unigenes

**Fig. 2:**
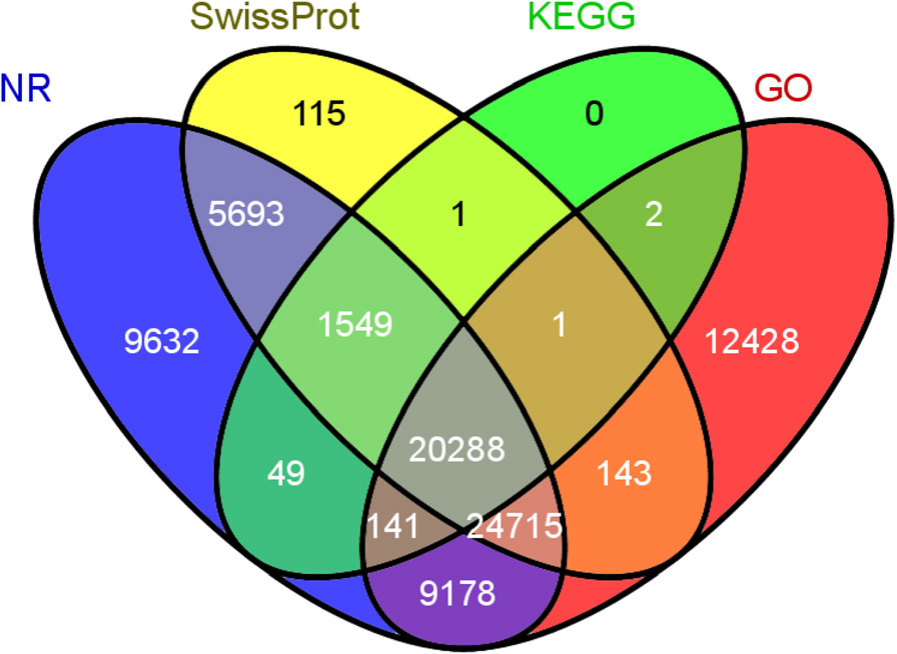
Venn diagram of the functional annotation. Venn diagram showing the number of unigenes blasted to the four databases: Nr, SwissProt, KEGG, and GO

All assembled unigenes were aligned against the public databases including non-redundant protein (Nr) database, non-redundant nucleotide (Nt) database, Pfam (Protein family), Swiss-Prot, Gene ontology (GO), Kyoto Encyclopedia of Genes and Genomes (KEGG) and Clusters of Orthologous Groups (KOG/COG). The number and percentage of unigenes annotated by each database is summarized in Table 3. A total of 20,288 unigenes were annotated in all four databases (Fig. 2). Notably, we found that 42.78 % of the unigenes were un-identified, which was common among previous studies performed using the *de novo* sequencing strategy (40.70 %, 62.48 %, and 45.73 %) [7, 19, 20]. Firstly, there is insufficient genome and EST information for *C. komarovii*. Secondly, the bioinformatics technical limitations including sequencing depth and read length are also the possible reason. Also, we found that the short sequences (200-500 bp) had a large percentage among the all unigenes (68.60 %).

**Table 3:** BLAST analysis of non-redundant unigenes against public databases

	Number of unigenes	Percentage (%)
Annotated in NR	71,243	47.9
Annotated in NT	21,807	14.66
Annotated in KO	29,737	19.99
Annotated in SwissProt	52,504	35.3
Annotated in PFAM	60,275	40.53
Annotated in GO	66,895	44.98
Annotated in KOG	38,709	26.02
Annotated in all Databases	8,566	5.76
Annotated in at least one Database	85,106	57.22
Total Unigenes	148,715	100

**Fig. 3:**
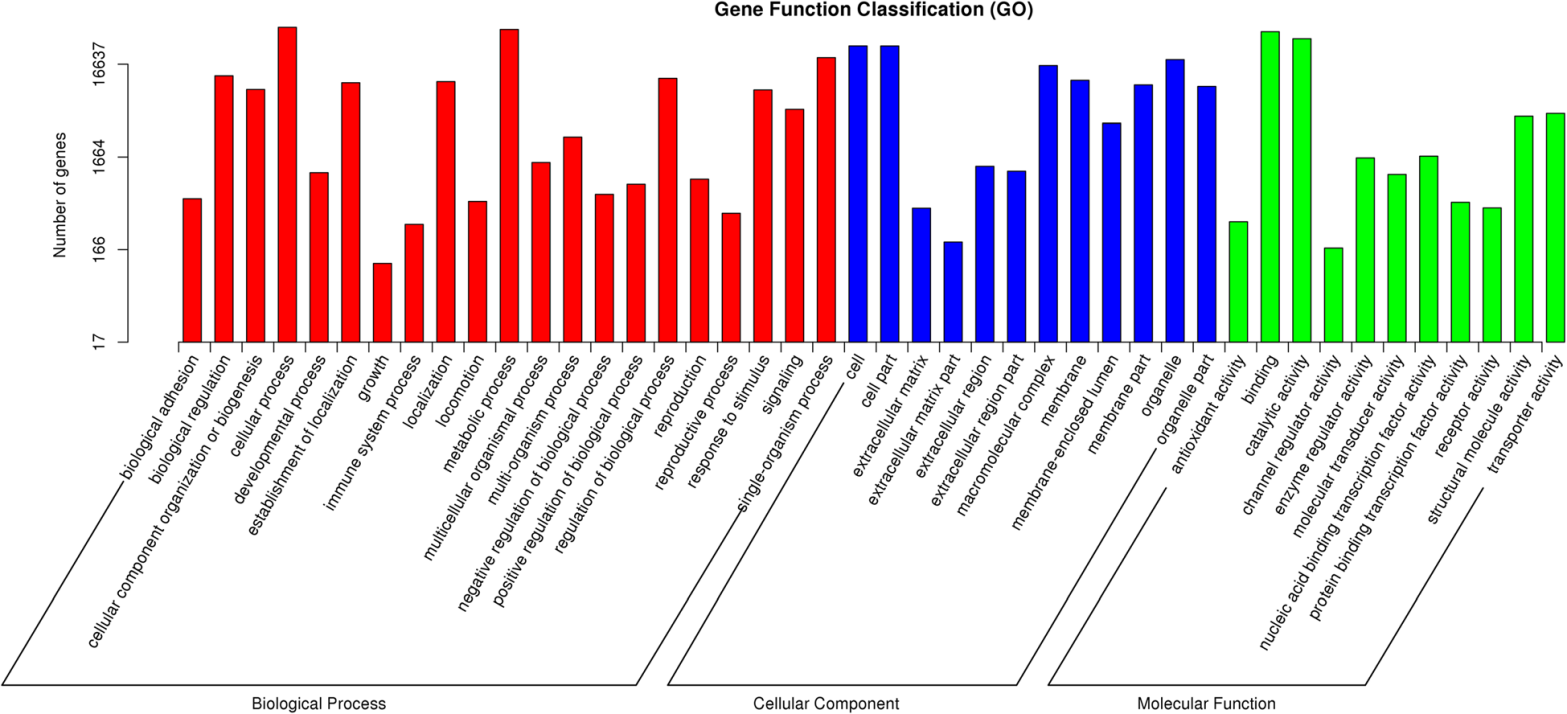
Histogram of gene ontology classification. The results are summarized in three main GO categories: biological process, cellular component and molecular function. The x-axis indicates the subcategories, and the y-axis indicates the numbers related to the total number of GO terms present; the unigene numbers that areassigned the same GO terms are indicated at the top of the bars

The GO terms for functional categorization was performed according to the Nr annotation. The main GO terms included biological process (BP), cellular component (CC), and molecular function (MF). Based on sequence homology, 66,895 unigenes were mainly categorized into 44 functional groups (Fig. 3, Additional file 1). The GO analysis indicated that a great number of identified genes were associated with various biological processes and molecular functions under drought. In the category of BP, the largest groups were cellular process and metabolic process. About 39,481 genes were annotated as the metabolic process category, which may allow the identification of novel genes involved in secondary metabolism pathways in drought acclimation. As for the MF category, unigenes with binding and catalytic activity formed the largest groups. For CC, the top three largest categories were cell, cell part, and organelle.

**Fig. 4:**
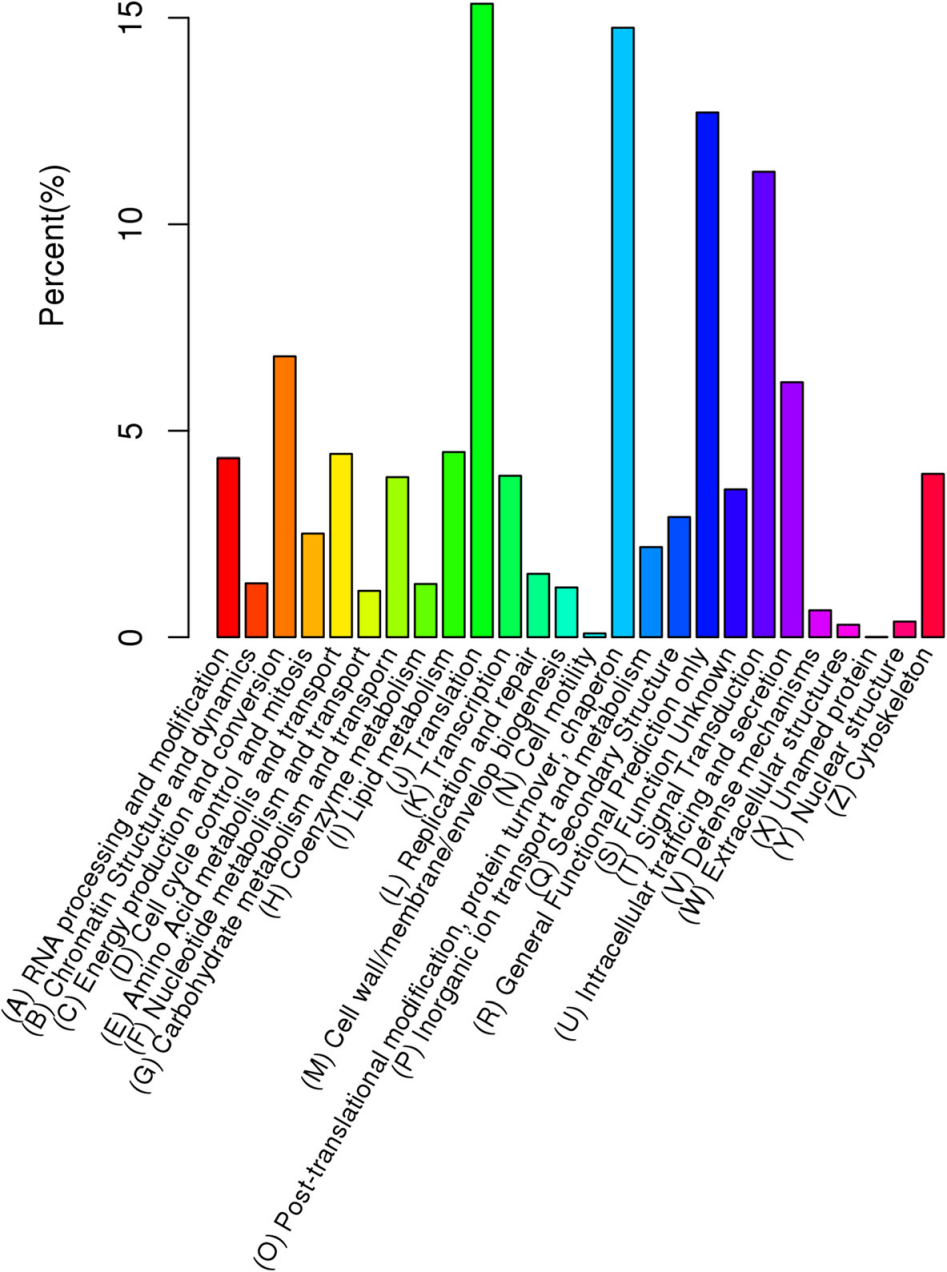
COG annotation of putative proteins. The unigenes were aligned to the COG database to predict and classify possible functions. Out of 71,243 Nr hits, 38,709 sequences were annotated and separated into 26 clusters

To further evaluate the reliability of our transcriptome results and the effectiveness of our annotation process, we searched the annotated sequences for genes with COG classifications. Out of 71,243 Nr hits, 38,709 sequences showed a COG classification (Fig. 4). Among the 26 COG categories, the cluster for “Translation” (5,939) represented the largest group, followed by “Post-translational modification, protein turnover, chaperon” (5,713), “General Functional Predication only” (4,921), and “Signal Transduction” (4,364). The categories “Cell motility” (35) was the smallest group.

**Fig. 5:**
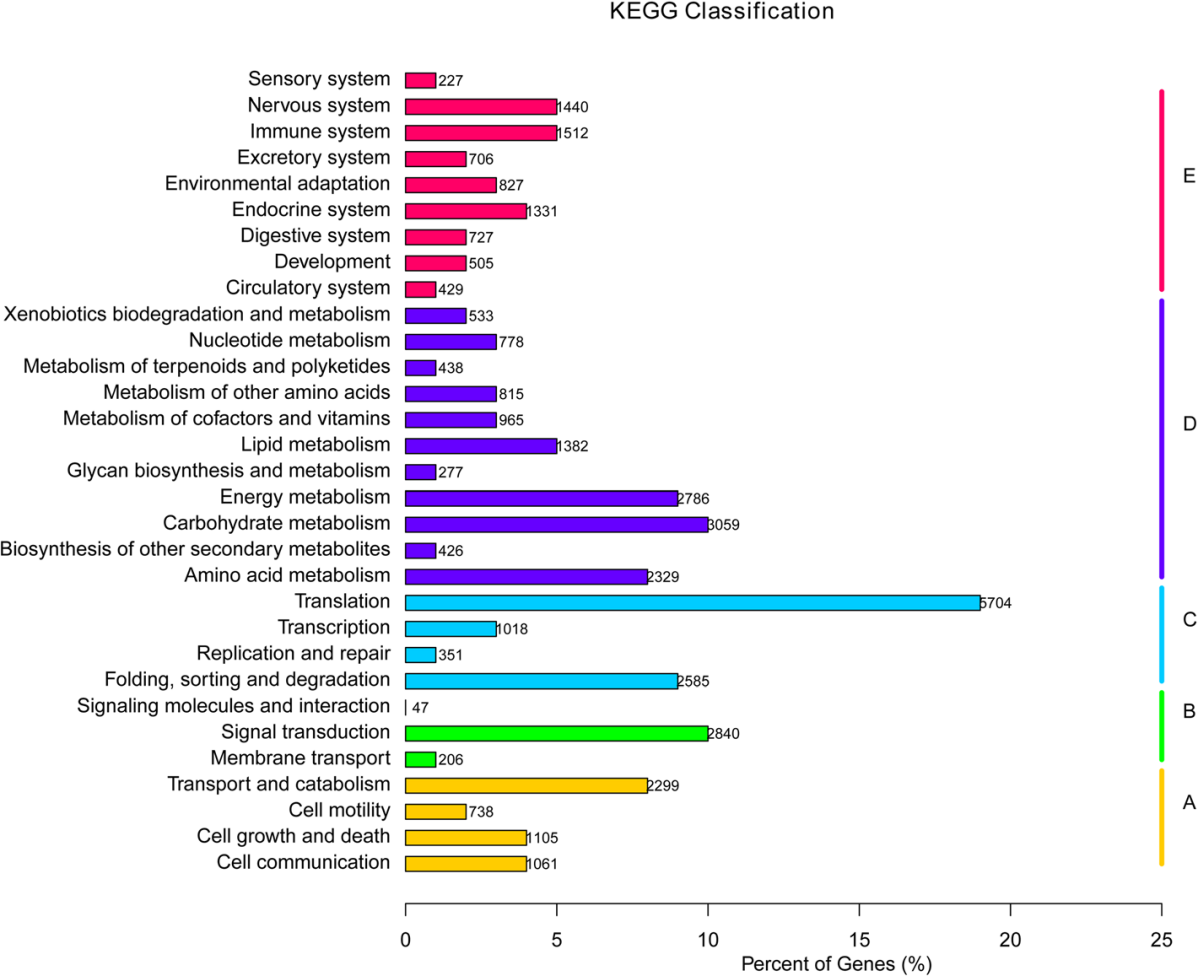
Pathway assignment based on KEGG. (**a**) Classification based on cellular process categories, (**b**) classification based on environmental information processing categories, (**c**) classification based on genetic information processing categories, (**d**) classification based on metabolism categories, and (**e**) classification based on organismal systems categories

To obtain a better understanding of the biological functions of the unigenes, we used the annotated sequences to search against the KEGG database. Among the 85,106 unigenes, 29,737 had significant matches and were assigned to 263 KEGG pathways (Fig. 5, Additional file 2). Of the 29,737 unigenes, the most strongly represented pathways were metabolism pathways. These annotations provide a resource for investigating the processes and pathways involved in responding to drought stress.

### Protein coding sequence prediction

All-unigenes were aligned against the protein databases and we obtained 72,537 coding sequences (CDS) from unigenes sequences and translated them into peptide sequences. Using the EST Scan, we assigned 54,145 unigenes CDS that could not be aligned to the protein databases and translated them into peptide sequences. Of these unigenes with CDS sequences, the majority (26,965) were over 500 bp and 11,868 were over 1000 bp. The transcriptome CDS predicted by BLASTx and ESTScan is shown in Additional file 3.

### DEGs among the drought stress

**Fig. 6:**
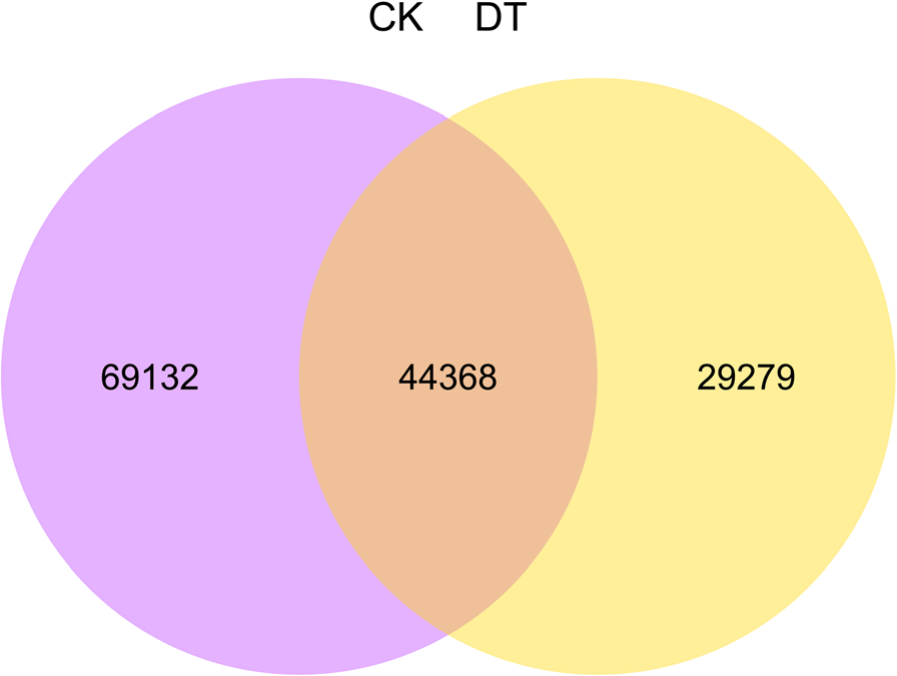
The Venn diagram of the specific genes under the drought stress. The Venn diagram showing the number of specific genes between the two treatments

**Fig. 7:**
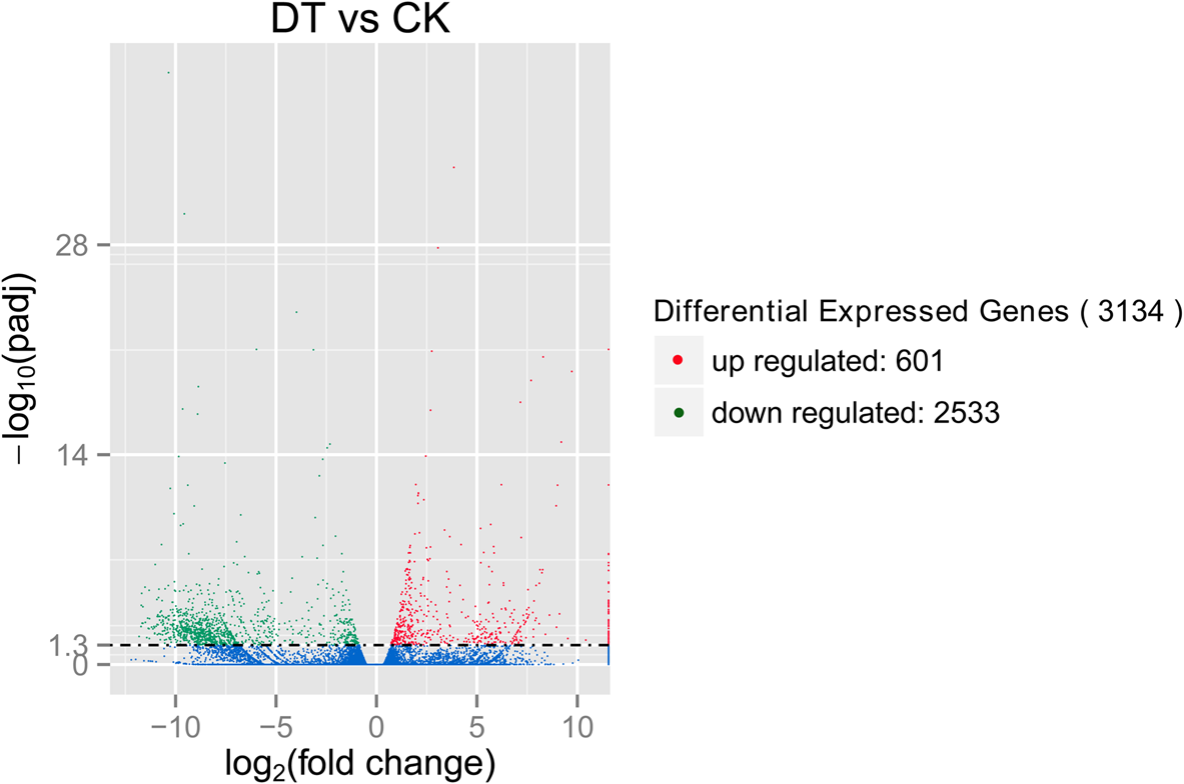
The distribution of DEGs. Red spots represent up-regulated DEGs and green spots indicate down-regulated DEGs. Those shown in blue are unigenes that did not show obvious changes. The volcano spots showed 3,134 unigenes including 601 unigenes up-regulated and 2,533 unigenes down-regulated were identified as DEGs

The analysis showed that a great number of unigenes were significantly affected upon treatment of *C. komarovii* seedlings with PEG solution. The Venn diagram showed the number of specific genes between the two treatments (Fig. 6). Also, we identified 3,134 unigenes as DEGs including 601 unigenes up-regulated and 2,533 unigenes down-regulated (Fig. 7, Additional file 4). The cluster analysis of DEGs between CK and DT samples were identified using a heat-map (Additional file 5).

### GO and KEGG enrichment analyses of DEGs

Enrichment analysis was performed to illustrate the biological functions of the identified DEGs. In total, 2,424 DEGs were enriched in 27 GO terms. We used up-regulated and down-regulated DEGs to perform GO enrichment analysis, respectively. Among the up regulated genes, three GO terms including “oxidation-reduction process” (120) and “single-organism metabolic process” (185) in the category of BP; “oxidoreductase activity” (113) in the category of MF were significantly enriched after drought treatments, indicating that genes in these processes may play pivotal roles in drought sensing (Additional file 6-A). Among the down regulated genes, GO terms including “protein metabolic process” (542), “cellular protein metabolic process” (437), “cellular component organization or biogenesis” (328), and “cellular response to stimulus” (265) in the category of BP; “macromolecular complex” (545) and “cytoplasm” (508) in the category of cellular component; “protein binding” (501) and “hydrolase activity” (484) in the category of MF were significantly enriched between DT and CK samples, suggesting that genes related to these processes may be suppressed in drought perception (Additional file 6-B). Furthermore, in the category of BP, DEGs with GO terms “cellular response to stimulus” suggesting drought treatment may have caused an efficient abiotic stress.

In total, 2,800 DEGs were enriched in KEGG pathways. The most enriched pathways were “metabolic pathways” (319), “Ribosome” (208), and “Biosynthesis of secondary metabolites” (154). We depicted the scatterplot comparing the KEGG pathway enrichment of the up regulated DEGs and down regulated DEGs, respectively (Additional files 7 and 8). In the KEGG pathway analyses, the genes involved in “cutin, suberine and wax biosynthesis” changed significantly, suggesting this pathway may play a vital role in protecting plants under drought treatment.

### Frequency and distribution of Simple Sequence Repeats

**Fig. 8:**
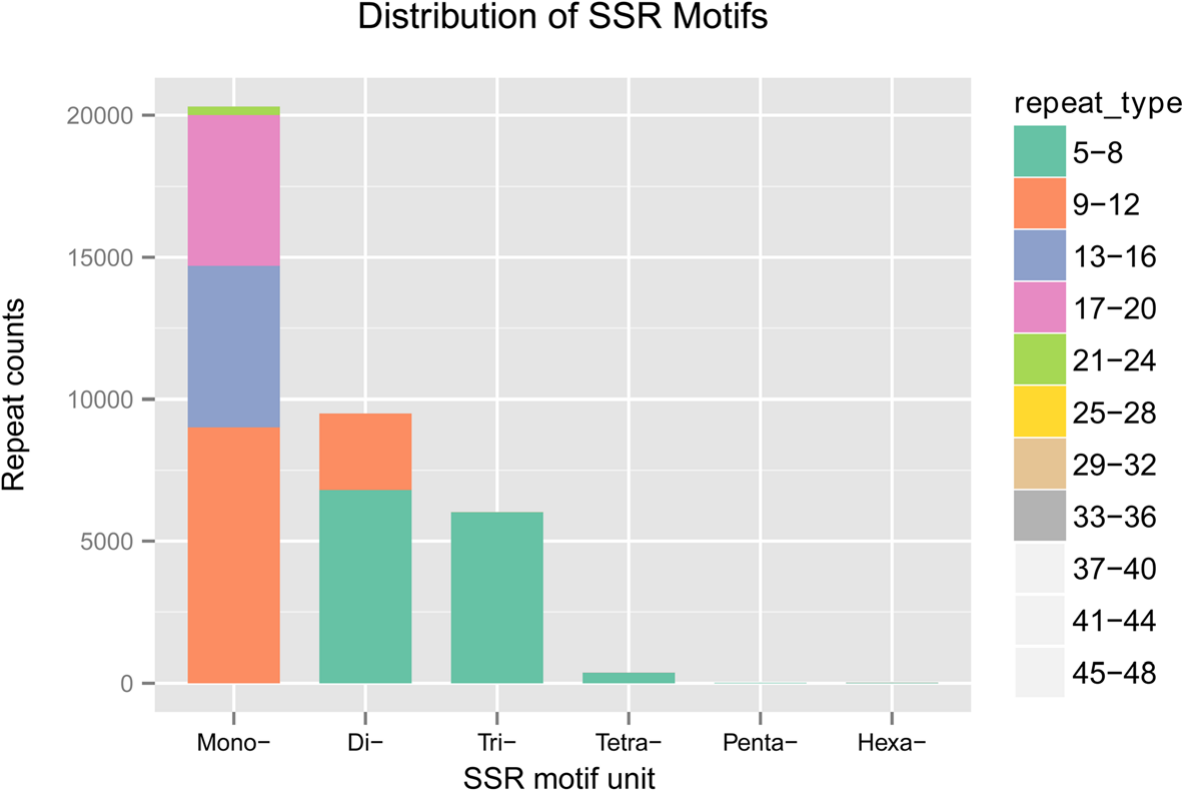
Frequency of EST-SSRs in *C. komarovii.* The x-axis indicates the repeat motifs of SSRs, and the y-axis indicates the total number of repeat counts. Color scale indicates the different type of SSRs. All SSRs based on the repeat motifs were divided into mono-nucleotide (20,304), di-nucleotide (9,505), tri-nucleotide (6,025), tetra-nucleotide (374), penta-nucleotide (15) and hexa-nucleotide (23), respectively

In genetics, a molecular marker is a fragment of DNA that is associated with a certain location within the genome, so it is important for gene mapping and molecular breeding. To develop new molecular markers, the unigenes generated in the *C. komarovii* transcriptome were used to mine potential microsatellites that were defined as di- to hexa-nucleotide motifs. Among the 148,715 examined sequences (89,843,452 bp total length) 36,246 SSRs were identified and the number of SSRs containing sequences reached 25,027. Additionally, 7855 sequences contained more than 1 SSRs. In the *C. komarovii* transcriptome, the SSRs frequency was 24.37 % and the distribution density was 2.48 per kb. In this study composed, all SSRs loci based on the repeat motifs were divided into mono-nucleotide (20,304), di-nucleotide (9,505), tri-nucleotide (6,025), tetra-nucleotide (374), penta-nucleotide (15) and hexa-nucleotide (23), respectively (Table 4, Fig. 8).

**Table 4:** Frequency of EST-SSRs in *C. komarovii*

Motif	Repeat numbers							Total	Percent
Length	5	6	7	8	9	10	>10		
Mono-	-	-	-	-	-	4,508	15,796	20,304	56.02 %
Di-	-	2,720	2,018	2,066	1,770	760	171	9,505	26.22 %
Tri-	3,427	1,794	768	31	-	-	5	6,025	16.62 %
Tetra-	328	41	1	-	2	1	1	374	1.03 %
Penta-	13	-	-	1	-	1	-	15	0.04 %
Hexa-	7	10	1	2	1	-	1	22	0.06 %
Total	3,775	4,565	2,788	2,100	1,773	5,270	15,974	36,245

We counted the frequency of SSRs with different numbers of tandem repeats and the results were shown in Table 5. The most abundant type was SSRs with six tandem repeats, followed by five tandem repeats, seven tandem repeats, eight tandem repeats, nine tandem repeats, ten tandem repeats, and more than 10 tandem repeats. The dinucleotide repeat motif AT/AT was the most abundant, followed by AG/CT, AAG/CTT, AAT/ATT, AC/GT, and AGC/CTG. The least repeat motif in SSRs was CG/CG (Table 5).

**Table 5:** Frequency of di- and tri-nucleotide EST-SSRs

Repeats	Repeat numbers							Total	(%)
Motif	5	6	7	8	9	10	>10		
AC/GT	-	356	175	127	84	59	49	850	5.47 %
AG/CT	-	752	511	377	301	230	73	2,244	14.45 %
AT/AT	-	1,589	1,326	1,562	1,385	471	49	6,382	41.09 %
CG/CG	-	23	6	-	-	-	-	29	0.19 %
AAC/GTT	243	119	59	4	-	-	-	425	2.74 %
AAG/CTT	833	479	184	4	-	-	2	1,502	9.67 %
AAT/ATT	569	380	151	3	-	-	-	1,103	7.10 %
ACC/GGT	247	98	59	4	-	-	1	409	2.63 %
ACG/CGT	66	29	7	1	-	-	-	103	0.66 %
ACT/AGT	116	46	15	3	-	-	-	180	1.16 %
AGC/CTG	477	225	84	1	-	-	-	787	5.07 %
AGG/CCT	296	135	75	5	-	-	1	512	3.30 %
ATC/ATG	398	228	110	4	-	-	1	741	4.77 %
CCG/CGG	182	55	24	2	-	-	-	263	1.69 %
Total	3,427	4,514	2,786	2,097	1,770	760	176	15,530	
(%)	22.07 %	29.07 %	17.94 %	13.50 %	11.40 %	4.89 %	1.13 %		

For each SSR the repeat unit, length and location were shown in Additional file 9. The results showed that 3’UTR SSRs (7,046) was the most abundant, followed by 5’ UTR SSRs (5,511) and CDS SSRs (1,412). The level of polymorphism detected by SSRs from different EST regions (CDS, 5’ UTR and 3’ UTR) varied across the different taxonomic levels. It suggested that 3’ UTR SSRs were more variable at the lower taxonomic levels (more related), the 5’ UTR SSRs had intermediate variability, and the CDS SSRs tend to differentiate at the higher taxonomic level [21]. To promote wide usage of the SSRs markers as a resource for gene mapping and molecular breeding, we designed primers for each of the SSRs (Additional file 9).

### Validation and expression pattern analysis

To further validate the reliability of the Illumina sequencing reads analysis, 13 candidate DEGs were selected and their expression profiles were compared between CK and DT samples using quantitative real-time (qRT) PCR. Among the chosen DEGs, CCAAT-binding nuclear transcription factor Y subunit B-3 (comp99898_c0), late embryogenesis abundant (LEA) proteins (comp115458_c2, comp110208_c0), zinc-dependent alcohol dehydrogenase (comp119655_c0), C3HC4 type RING finger (comp118993_c0), R2R3-MYB transcription factor (comp118180_c2), osmotic stress-induced zinc-finger protein (comp115030_c0), heat-shock proteins 70 (HSPs) (comp117600_c5), ethylene- responsive transcription factor (comp114730_c4), WRKY1b transcription factor (comp107172_c1), nitrate transporter (comp111651_c0), homeobox leucine zipper protein (comp106817_c0), WAX2-like protein (comp117767_c0) have been known to be related to responding to abiotic stresses. The expression pattern of all these DEGs obtained through qRT-PCR data were consistent with High-throughput RNA-sequencing (RNA-Seq) data, confirming that the Illumina results were reliable (Table 6).

**Table 6:** Real-time RT-PCR with putative unigenes associating with drought stress

Gene ID	Gene annotations	Relative gene expression by qRT-PCR(2-^ΔΔCT^)	Expression difference analysis of Illumina(log_2_FoldChange)
Comp99898_c0	Nuclear transcription factor Y subunit B-3	3.93 ± 1.03	1.8075
Comp115458_c2	Late embryogenesis abundant protein 4	111.98 ± 5.33	5.9312
Comp119655_c0	Zinc-dependent alcohol dehydrogenase	4.28 ± 0.92	7.8852
Comp118993_c0	Zinc finger, C3HC4 type	58.82 ± 0.91	7.1409
Comp110208_c0	Late embryogenesis abundant group 1	6.43 ± 1.30	6.0027
Comp118180_c2	R2R3-MYB transcription factor	6.30 ± 0.10	5.6962
Comp115030_c0	Osmotic stress-induced zinc-finger protein	22.24 ± 4.34	3.7410
Comp117600_c5	Heat-shock protein 70	6.36 ± 0.89	2.0935
Comp114730_c4	Ethylene-responsive transcription factor	1.47 ± 0.30	1.6833
Comp107172_c1	WRKY1b transcription factor	6.39 ± 2.03	1.9783
Comp111651_c0	Nitrate transporter	11.62 ± 0.73	3.9292
Comp106817_c0	Homeobox leucine zipper protein	8.67 ± 2.03	2.5621
Comp117767_c0	WAX2-like protein	4.57 ± 1.51	1.6953

### DEGs involved in signaling transduction and regulation in *C. komarovii* underlying drought stress

Regulatory genes identified in transcriptome are mainly involved in signaling transduction pathways and in controlling the expression of functional genes in stress responses, which can be divided into three categories: ionic and osmotic homeostasis signaling, detoxification response, and growth regulation pathways [22]. DEGs involved in signaling transduction and regulation are listed in Table 7.

**Table 7:** Overview of DEGs involved in functional and regulatory proteins during drought stress

Functional proteins	Number	Regulatory proteins	Number
Protection factors of macromolecules		Ion signaling	
Heat shock protein	24	Calcium signaling pathway	21
Flavonoids biosynthesis	10		
LEA protein	5	Protein kinase, phosphatases for osmotic stress	
Universal stress protein	4	MAPK signaling pathway	44
Osmotin	3	Protein phosphatase	40
Water channels, Transporters		Osmotic stress-activated phospholipid signaling	
Transporter	20	Diacylglycerol	6
Aquaporin	12	Phospholipase C	4
Osmolyte biosynthesis		Detoxification signaling	
Proline	20	Heavy Metal Transport/Detoxification Protein	4
Trehalose	3		
Sucrose	2	Transcription factor	
Raffinose	1	C3HC4-type RING finger	19
Betaine	1	MYB	11
Mannitol	1	bZIP	9
		NAC	8
Detoxification enzymes group		CBF/NF-Y	7
Glutathione S-transferase	22	C2H2-type RING finger	7
Superoxide dismutase	4	PHD-finger	7
Soluble epoxide hydrolase	2	WRKY	4
Catalase	1	bHLH	4
Ascorbic peroxidase	1	ERF/AP2	1

In the ionic signaling pathway, calcium signaling has been implicated in plant responses to a range of abiotic stresses including chilling, drought, salinity, heat shock, oxidative stress, anoxia, mechanical perturbation [23]. In this study, 21 DEGs encoding Ca^2+^ binding proteins were detected in the *C. komarovii* transcriptome. Stress-induced changes in the cytosolic concentration of calcium ion is thought to be the primary stimulus-sensing signal that is transduced via calmodulins (CaMs), the calcium/calmodulin-dependent protein kinases (CaM kinase/CDPKs), the calcineurin B-like proteins (CBLs) and other calcium binding proteins to effect the downstream responses involved in the protection of the plant and adaptation to the new environmental stimuli [24, 25].

Osmotic stresses activate several protein kinases and phosphatases mediating osmotic homeostasis or detoxification responses [22]. Here we detected 44 DEGs involved in mitogen-activated protein kinase (MAPK) signaling pathway and 40 DEGs encoding phosphatase in the *C. komarovii* transcriptome. MAPK cascades, as a major plant transduction components, regulate numerous processes relaying downstream of receptors or sensors and transduce environmental and developmental signals into adaptive and programmed responses [26, 27]. Meanwhile, the protein phosphatases also have function in stress signaling. In *C. komarovii,* we identified a salt-sensitive 3’-phosphoadenosine-5’-phosphatase HAL2/SAL1 (comp115181), which had the same transcript profile with phosphatases SAL1 in *Arabidopsis* [28]. This may play a role in cellular retrograde signaling pathways during the drought stress.

Upon drought stress, changes in phospholipid composition are detected in plants and membrane phospholipids can activate groups of phospholipases that catalyze the formation of multiple second-messenger molecules [29]. In this pathway, 6 diacylglycerol (DAG) and 4 phospholipase C (PLC) are detected, which further play important roles during stress responses [22].

Transcription factors (TFs) are key regulators of plants, which play essential roles in regulating responses to diverse biotic/abiotic stresses and activating the down-stream targets to improve the stress tolerance of plants [6]. The most abundant TFs families detected among DEGs in the *C. komarovii* transcriptome were C3HC4-type RING finger, MYB, bZIP, and NAC. Meanwhile, TFs families such as CBF/NF-Y, C2H2-type RING finger, PHD-finger, WRKY, bHLH, GRAS, MYC, and ERF/AP2 were also observed (Table 7). All these families have been known to be previously acting in enhancing drought tolerance and improving water-use efficiency. Thus, differentially expression of these TFs may affect the expression of functional genes in response to drought stress [30, 31].

### DEGs associated with the major functional groups in *C. komarovii* during drought stress

During the environmental stresses, plants activate many functional genes that directly protect plants and improve tolerance to adapt adverse environment. Functional genes group that can be comprised of several categories: protection factors of macromolecules (HSPs, LEA proteins, flavonoids, osmotin, and universal stress protein, etc.), membrane related protein (water channels and transporters, etc.), osmolyte biosynthesis (proline, betaine and sugars, etc.), and detoxification enzymes (glutathione S-transferase, superoxide dismutase, soluble epoxide hydrolase, catalase, and ascorbic peroxidase, etc.) [30]. DEGs associated with the major functional groups are listed in Table 7.

HSPs are environmentally induced proteins that have functions in acquired stress tolerance and enable plants to cope with heat and other environmental stresses, also play a role via operating together with other stress-response mechanisms and functional components, collaborating in decreasing cellular damage [32]. In this study, 24 DEGs detected in the *C. komarovii* transcriptome were members of the HSPs family and these genes showed significant changes at DT group under drought treatment. Flavonoids are ubiquitous plant secondary metabolites with diverse functions in growth, development, reproduction, and adaptation to environmental stresses [33]. Exposed to the abiotic stresses, genes involved in the flavonoid biosynthesis pathway are up-regulated and the flavonoids accumulated rapidly. In this study, the DEGs encoding flavanone 3-hydroxylase (F3H), flavonoid 3’, 5’-dydroxylase (F3'5'H), flavonoid 3’-monooxygenase (FM), and UDP-glucose flavonoid glucosyltransferase (UFGT) were up-regulated and could also play a protective role in response to drought stress. Comp115458 and comp110208 were the members of the LEA proteins family with high expression under drought conditions (nearly 6-fold up-regulation). In different plant species, many *LEA* genes have been identified and demonstrated to be related to tolerance against water deficiency, osmotic, salt, and cold stresses [34]. In addition, several functional proteins such as universal stress protein (USP) and osmotin were also detected. Up-regulation of all these protection factors may protect *C. komarovii* from the stress and lead to enhance tolerance to drought stress*.*

Drought stress may influence the expression levels of genes encoding water channels and transport related proteins. A total of 20 DEGs were annotated as transporter including ABC transporter (12 DEGs), zinc transporter (1 DEGs), sugar transporter (3 DEGs), and nitrate transporter (4 DEGs), which were significantly affected by abiotic stresses. In *Arabidopsis*, the function of ABCG25, ZTP29, and NRT1.1 in stomatal opening, regulation of ion channels, and response to stresses have been well studied [35–37]. Aquaporins act as water channels, which play a vital role in plant water relations and response to drought. In this study, all of the 12 DEGs encoding aquaporins were down-regulated, which may be a mechanism to reduce membrane water permeability and to allow cellular water conservation during drought stress [38].

To prevent water loss from the cell and protect proteins, plants as accumulate organic osmolytes, including proline, trehalose, sucrose, raffinose, betaine, and mannitol [5]. In previous studies, the contents of betaine, starch, proline, and soluble sugar (i.e., fructose, sucrose, and trehalose) shown significant increases in *C. komarovii* under drought stress. In this study, 28 DEGs involved in biosynthetic pathways for these osmolytes, suggesting that the increased concentration of these osmolytes play an important role in maintaining cell turgor and conserving water in acute stress.

Detoxification enzymes such as glutathione S-transferase, superoxide dismutase, soluble epoxide hydrolase, catalase, glutathione peroxidase, and ascorbate peroxidase are involved in protection of cells against reactive oxygen species [39]. In this study, there were 30 DEGs detected belonging to detoxification enzymes group. Interestingly, the results indicated that most of these genes were down-regulated during drought stress. Several studies have reported that the activity and expression level of the detoxification enzymes can be induced by the drought stress. However, this induction would be damaged inevitably under heavy stresses conditions, suggesting that drought treatment might lead a severe impact on the detoxification enzymes systems.

### DEGs related to formation of plant cuticle under drought stress

**Fig. 9:**
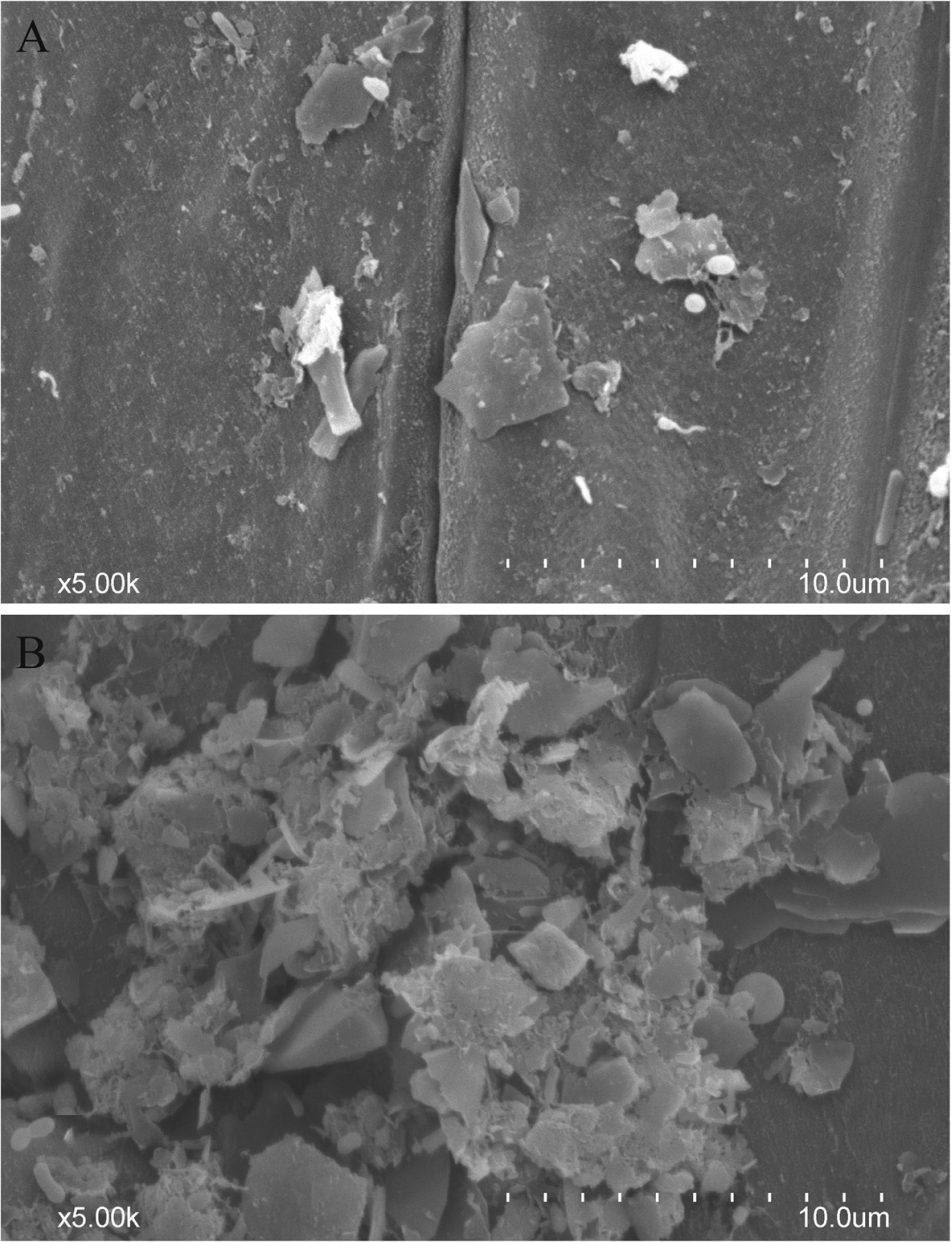
The SEM views of root cuticular wax. SEM images of root surface of control group (**a**) and drought treatment group (**b**). Scale bars in A and B are 10 μm

**Fig. 10:**
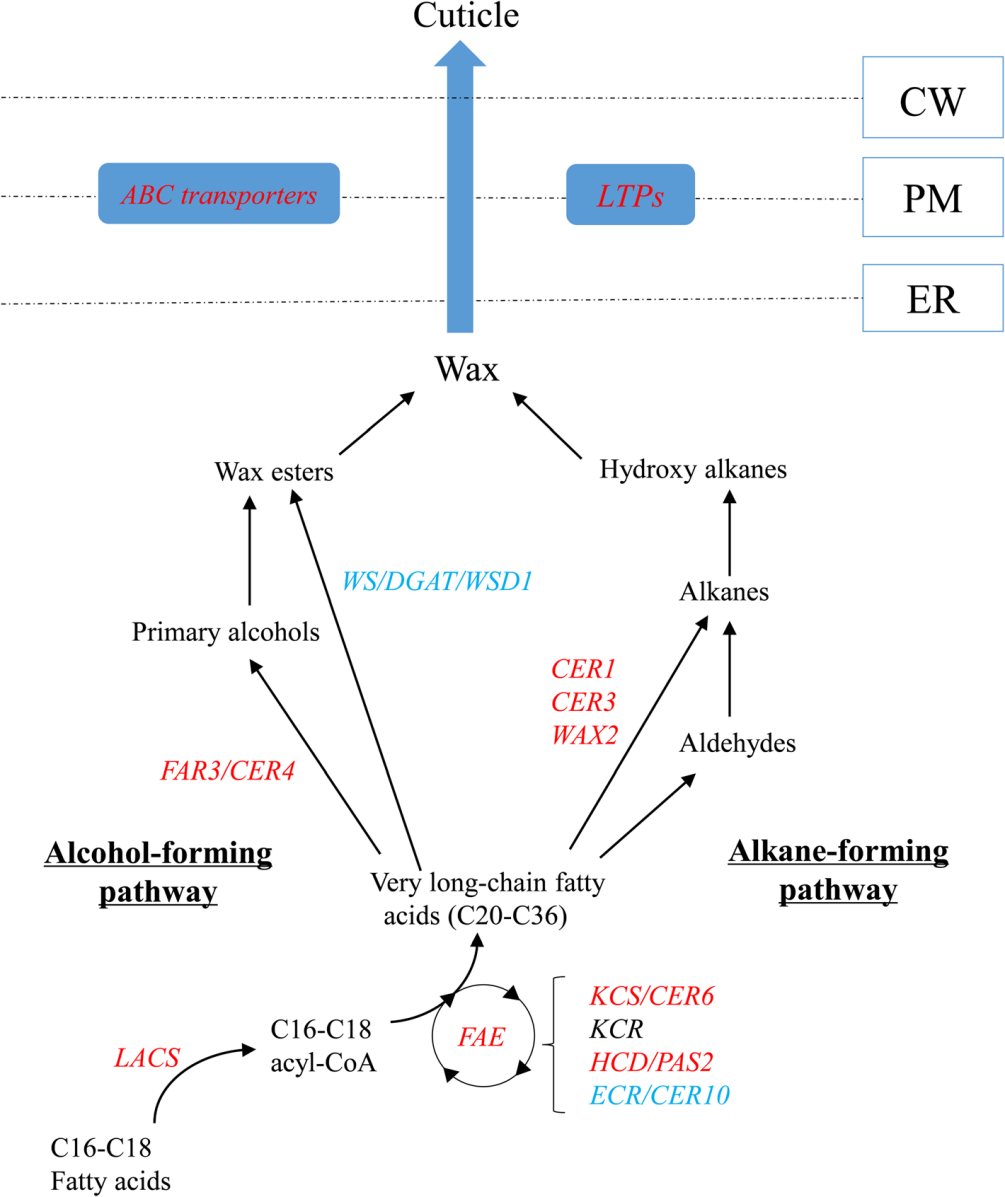
Schematic representation of cuticular wax biosynthesis and export. Cuticle biosynthesis pathway, which are composed of three stages: In the first stage, C16 and C18 fatty acids are generated by *de novo* synthesis. The second stage involves the elongation of C16 and C18 fatty acids into VLCFAs composed of C20 to C36. In the third stage, VLCFAs are modified into the major wax products according to two distinct biosynthetic pathways: the alcohol-forming pathway and the alkane-forming pathway. Wax products are mobilized from endoplasmic reticulum (ER) through the plasma membrane (PM) to the outside of the cell wall (CW) by ABC transporters and lipid transfer proteins (LTPs). Genes in red displayed significant variation of expression. Genes in blue were only detected in CK samples, and gene in black was not detected in both samples

Plant cuticle, which can be divided into the inner cutin and outer wax, is the hydrophobic protection layer against water loss and protects plants from the deleterious effects of light, temperature, osmotic stress, high salinity, and physical damage [40, 41]. When *Arabidopsis* is subjected to abiotic stresses (i.e., water deficit, NaCl, or ABA treatments), shown a significant accumulation in leaf cuticle lipids and these stress treatments led to higher amount in wax and alkanes. However, the increases in total cutin monomer amount (by 65 %) and leaf cuticle thickness (49 %) were only observed under water deficit condition [42]. Furthermore, the views of the scanning electron microscopy (SEM) showed the increase in accumulation of wax under drought stress (Fig. 9). It suggested that plant cuticle played a vital role in response to abiotic stresses, especially drought stress and increased amount of cuticle had been associated with improved drought tolerance [42]. The cuticle-associated genes involved in the biosynthesis, export, and regulation were highly induced by environmental stresses [43]. In this study, DEGs related to plant cuticle had been identified, which can be classified into: biosynthesis, export, and regulation (Table 8, Fig. 10).

**Table 8:** Summary of DEGs encoding proteins related to formation of plant cuticle under drought stress

	Gene ID	Fold change	Name	Annotations	Function
Biosynthesis	Comp107927_c1	+1.1	ACC1	Acetyl-CoA carboxylase 1	Formation of malonyl-CoA
	Comp52056_c0	+1.5	FAS	Fatty acid synthase complex	Synthesis *de novo* of acyl chains
	Comp136331_c0	-	ACP	Acyl carrier protein	Synthesis *de novo* of acyl chains
	Comp52056_c0	+1.5	FAE1	Long chain fatty acid elongase	VLCFA synthesis
	Comp114759_c0	+1.8	KCS6/CER6/CUT1	β-ketoacyl-CoA synthase	VLCFA synthesis
	Comp52476_c0	+1.2	HCD/PAS2	β-hydroxyacyl-CoA dehydrogenase	VLCFA synthesis
	Comp105753_c0	+1.7	HCD/PAS2	β-hydroxyacyl-CoA dehydrogenase	VLCFA synthesis
	Comp105260_c0	+6.9	HCD/PAS2	β-hydroxyacyl-CoA dehydrogenase	VLCFA synthesis
	Comp104441_c0	-	ECR/CER10	Trans-2-enoyl-CoA reductase	VLCFA synthesis
	Comp105777_c0	+1.6	CER4/FAR3	Alcohol-forming fatty acyl-CoA reductase 3	Primary alcohol synthesis
	Comp114784_c0	+3.7	CER4/FAR3	Alcohol-forming fatty acyl-CoA reductase 3	Primary alcohol synthesis
	Comp115904_c0	-	WS/DGAT/WSD1	Wax synthase/Diacylglycerol acyltransferase	Primary alcohol synthesis
	Comp117767_c0	+1.7	CER1/CER3/WAX2	ECERIFERUM 1	Alkane synthesis
	Comp98688_c0	+1.0	GPAT4	Glycerol-3-phosphate acyltransferase	Cutin and suberin synthesis
	Comp113958_c1	+1.2	GPAT6	Glycerol-3-phosphate acyltransferase	Cutin and suberin synthesis
	Comp70862_c0	+1.4	LACS4	Long chain acyl-CoA synthetase	Wax and cutin synthesis
	Comp118110_c0	+1.5	ATT1	Cytochrome P450 CYP86A2	Cutin synthesis
	Comp98682_c0	+1.8	HORST	Cytochrome P450 CYP86A1	Suberin monomer synthesis
	Comp74052_c0	+1.3	GDSL	GDSL-like Lipase	Cutin deposition
	Comp73265_c0	+1.3	GDSL	GDSL-like Lipase	Cutin deposition
	Comp92130_c0	+2.1	GDSL	GDSL-like Lipase	Cutin deposition
Export	Comp118089_c0	+1.3	ABCG32/WBC	ABC transporter G family member	Export of wax and cutin molecules
	Comp98900_c2	+1.4	LTPs	Lipid transfer proteins	Wax extracellular transport
	Comp52067_c0	+1.0	nsLTPs	Non-specific lipid transfer proteins	Wax extracellular transport
	Comp120307_c0	+1.0	nsLTPs	Non-specific lipid transfer proteins	Wax extracellular transport
	Comp86599_c0	+2.1	nsLTPs	Non-specific lipid transfer proteins	Wax extracellular transport
	Comp74939_c0	+4.3	nsLTPs	Non-specific lipid transfer proteins	Wax extracellular transport
Regulation	Comp114730_c4	+1.7	WIN1/SHINE1	Ethylene responsive transcription factor	Regulate cuticle development

The first category encoding proteins involved in cuticle biosynthesis pathway, which are composed of three stages. In the first stage, the major precursors of all cuticle aliphatic components C16 and C18 fatty acids are generated by *de novo* synthesis in plastids [43]. During this process, the multifunctional acetyl-CoA carboxylase (ACCase) catalyze biosynthesis of malonyl-CoA from acetyl-CoA. Then *de novo* synthesis of acyl chains in plastids catalyzed by fatty acid synthases (FAS) complex, which includes acyl carrier protein (ACP) as a cofactor [41]. In the second stage, C16 and C18 fatty acids are further elongated into very long chain fatty acids (VLCFAs) composed of 20 to 36 carbons [43]. The biosynthesis of VLCFAs is catalyzed by the microsomal elongase, a multi-enzyme complex, called fatty acid elongase (FAE), which involves four serial enzyme reactions: condensation by a β-ketoacyl-CoA synthase (KCS), reduction of β-ketoacyl-CoA by a β-ketoacyl-CoA reductase (KCR), dehydration of β-hydroxyacyl-CoA by a β-hydroxyacyl-CoA dehydratase (HCD), and additional reduction of enoyl-CoA to a C2 unit extended acyl-CoA by enoyl-CoA reductase (ECR) [44]. In the present study, all enzymes except KCR were detected among DEGs and the expression rates of these DEGs increased nearly 1.0-7.0 fold under drought condition. However, nine ECRs (not all shown in Table 8) were only detected in the CK groups. The KCS has substrate specificities, nevertheless, the KCR1, HCD/PAS2, and ECR/CER10 could participate in all elongation cycles. It suggests that down-regulation of ECRs may not affect the formation of VLCFAs during this process [44]. In the third stage, VLCFAs are modified into the major wax products, including alcohols, esters, aldehydes, alkanes, and ketones [43]. In the alcohol-forming pathway, two putative fatty acyl-CoA reductase 3 (FAR3/CER4) were highly induced (nearly 1.6 and 3.7 fold up-regulation) by drought stress. However, wax synthase/diacylglycerol acyltransferase (WS/DGAT) encoding gene WSD1 was only detected in CK group. In the alkane-forming pathway, a DEG annotated as ECRIFERUM 1 (CER1) and WAX2-like protein (WAX2) was detected. In *Arabidopsis*, a multiprotein enzyme complex including CER1, CER3/WAX2/YRE are core components of a very long chain alkane synthesis complex, which plays an important role in wax alkane synthesis [45]. Furthermore, DEGs encoding glycerol-3-phosphate acyltransferase (GPAT), long chain acyl-CoA synthetase 4 (LACS4), cytochrome P450 CYP86A1 (HORST), CYP86A2 (ATT1), and GDSL-like lipase were also detected and responsive to drought stress, which have been involved in suberin, cutin and wax synthesis in previous studies [46–50].

The second category encoding proteins involved in export of wax and cutin cuticle from endoplasmic reticulum (ER) through the plasma membrane (PM) to the outside of the cell [43]. ATP BINDING CASSETTEG32 (ABCG32), an ABC transporter from the pleiotropic drug resistance family is required for exporting wax compounds in *Arabidopsis* [51]. In this study, a DEG annotated as ABCG32 was detected, suggesting that the exporting process of wax and cutin molecules might be carried by ABCG32. During the transport of cuticular lipids through the cell wall, the lipid transfer proteins (LTPs) were involved in this process. The experimental evidence indicates that non-specific LTPs (nsLTPs) have function in binding a wide variety of acyl chains, including cutin monomers. In addition, the expression of LTPs is significantly up-regulated under drought and ABA mediated stresses [52]. In this study, 5 DEGs encoding LTPs were annotated and up-regulated (nearly 1.0 and 4.3 fold), which suggested these wax-associated LTPs might play an important role for wax extracellular transport and accumulation in *C. komarovii*.

The third category encoding proteins have function in regulation of cuticle biosynthesis and transport. In *Arabidopsis*, ethylene responsive transcription factors (AP2/ERFs) have been widely shown involvement in various developmental processes, especially regulation of cuticle biosynthesis, accumulation and transport in responses to abiotic stresses [43]. The WAX INDUCER 1 (WIN1) and SHINE 1 (SHN1) belonging to ERF subfamily have been identified and shown preferentially expressed in roots and floral tissues. Overexpression of SHINE1 had an increasing cuticular wax accumulation in transgenic plants than wild-type plants which improved drought stress tolerance in *Arabidopsis* [53]. The DEG identified as AP2/ERFs in the *C. komarovii* transcriptome might have remarkable function as a coordinator of cuticle biosynthesis gene expression.

## Conclusions

*C. komarovii* is a xerophytic plant species, which possesses strong resistance to drought stresses. Here, we generated a transcriptome sequencing of *C. komarovii* root tissues using a NGS technology and performed comprehensive analysis of the drought-responsive genes and explored the mechanisms of drought-tolerance. The results showed that DEGs encoding regulatory and functional proteins had different expressions under drought stress. Importantly, the DEGs were involved in biosynthesis, export, and regulation of plant cuticle, suggesting that plant cuticle may play a vital role in response to drought stresses and accumulation of cuticle may allow *C. komarovii* to improve the tolerance to drought stress. In conclusion, our comprehensive transcriptome analysis will provide valuable resource for further investigation into the molecular mechanisms of desert plants under drought condition and facilitate the exploration of drought-tolerant candidate genes.

## Methods

### Ethics

This research did not involve any human subjects, human material, or human data. *C. komarovii* in current research did not belong to the endangered or protected species.

### Plant materials and drought treatment

The seeds of *C. komarovii* were collected from Yinchuan City, Ningxia Autonomous Regions, and China. After surface sterilization, the seeds of *C. komarovii* were planted in pots containing soil and vermiculite (with 2:1 ratio of soil to vermiculite) under the following conditions: natural light, photoperiod 16 h light/8 h darkness, day/night temperature of 28 °C/16 °C. So we selected the 4-weekold seedling for sampling and gene expression analysis. Drought treatments were applied to the 4-week-old plants, the plants were extracted from pots and the soil washed off with deionized water, then cultivated in half strength of Hoagland medium solution. The plantlets were classified into two groups: control sample (CK) and drought-treatment (DT), and each group was performed with two independent biological replicates (DT_1, DT_2, CK_1, and CK_2). For drought treatment, 20 % PEG6000 was added to the Hoagland medium solution. The root tissues of each sample from the control and drought-treatment plants were harvested after 72 h and immediately frozen in liquid nitrogen and stored at -80 °C till use.

### RNA extraction and quality determination

Total RNA were obtained from two different biological replicates for each group sample using TRIzol Reagent (Invitrogen) following the manufacture’s protocol. The RNA quality was evaluated using Bioanalyzer 2100 (Agilent Technologies, CA, USA); the A260/A280 ratios of both samples from 2.0 to 2.1. The best RNA samples having RIN above 8.0 were chosen for cDNA library preparation.

### cDNA library preparation for transcriptome sequencing

A total amount of 3 μg RNA per sample was used as input material for the RNA sample preparations. Sequencing libraries were generated using NEBNext® Ultra™ RNA Library Prep Kit for Illumina® (NEB, USA) following manufacturer’s recommendations. Briefly, mRNA was purified from total RNA using polyT oligo-attached magnetic beads. First strand cDNA was synthesized using random hexamer primer and M-MuLV Reverse Transcriptase. Second strand cDNA synthesis was subsequently performed using DNA polymerase I and RNase H. In order to select cDNA fragments of preferentially 150 ~ 200 bp in length, the library fragments were purified with AMPure XP system (Beckman Coulter, Beverly, USA) and library quality was assessed on the Agilent Bioanalyzer 2100 system. After cluster generation, four libraries (CK_1, CK_2, DT_1 and DT_2) preparations were sequenced on the Illumina Hiseq^™^ 2000 platform to generate 100 bp paired-end reads (Novogene, China).

### Quality control, transcriptome assembly and functional annotation

Clean reads were obtained by removing adaptor sequences, unknown sequences ‘N’, low quality reads from raw data. At the same time, Q20, Q30, GC-content and sequence duplication level of the clean data were calculated. All the analyses were based on clean reads with high quality. Transcriptome de novo assembly was carried out using Trinity [54] with the default settings, except that min_kmer_cov set to 2 by default. To assign predicted gene descriptions for the assembled unigenes, they were aligned against the plant protein dataset of Nr, Nt, Pfam, Swiss-Prot/Uniprot protein database, and KOG/COG, respectively, using BLASTX with a significance threshold of *E*-value <10^-5^. For sequences aside from those obtained from BLASTX searches, we used the EST Scan program to determine the sequence orientation. The GO terms for functional categorization was analyzed using Blast2go software [55]. The pathway assignments were carried out by sequence searches against the KEGG database, also using the BLASTX algorithm with an *E*-value threshold of 10^-5^.

### Polymorphism detection

SSRs of the transcriptome were identified using the MISA (http://pgrc.ipk-gatersleben.de/misa/misa.html). The minimum number of nucleotide repeats specified during SSR analysis was 20, 10, 7, 5, 5, and 5 for mono-, di-, tri-, tetra-, penta-, and hexa-nucleotide repeats, respectively. The maximum number of bases interrupting 2 SSRs in a compound microsatellite was set at 100 bp. The primer for each SSR was designed using Primer3 program with the default parameter (http://primer3.sourceforge.net/releases.php).

### Differential expression analysis of unigenes

Differential expression analysis of two groups was performed using the DEGseq R package (1.10.1) [56]. DEGseq provide statistical routines for determining differential expression in digital gene expression data using a model based on the negative binomial distribution. The resulting *P* values were adjusted using the Benjamini and Hochberg’s approach [57] for controlling the FDR (false discovery rate). In this analysis, the genes with a threshold *P*-value <0.001 and the absolute value of log_2_Ratio (DT/CK) >1 screened by DEGseq were assigned as differentially expressed.

Functional enrichment analysis including GO and KEGG enrichment analysis of the DEGs was implemented by the GOseq R packages [58] based on Wallenius non-central hyper-geometric distribution [59]. We calculated the numbers of all DEGs, up regulated and down regulated genes to each GO term, respectively. We used KOBAS software [60] to test the statistical enrichment of DEGs in KEGG pathways.

### Validation and expression pattern analysis

To experimentally validate the transcriptional abundance results from sequencing and computational analysis, 13 unigenes were selected for qRT-PCR analysis. 2 μg total RNA was reverse transcribed to first-strand cDNA using the high-capacity RNA-to-cDNA kit (Applied Biosystems, Foster City, CA) according to manufacturer’s specifications. Diluted cDNA was used as template in each well for the qRT-PCR analysis. Primers were designed using Primer Premier 5 and listed in Additional file 10. The figure of an agarose gel with PCR products was shown in Additional file 11. The qRT-PCR was performed by 20 μL reaction mixture in 96-well plate with ABI7500 Fast Real-Time PCR System, using SYBR *Premix Ex Taq™ II kit* (Takara). Reactions were performed at 95 °C for 30s, 40 cycles of 95 °C for 3 s, and 60 °C for 34 s. Each sample was performed with two independent biological replicates and each biological replicate had three technical replicates. Statistical analysis of the relative expression levels of each gene normalized to *EF-1-α* [61] was calculated using comparative Ct value method.

### SEM Analysis

To view cuticular waxes, sections of roots were treated in 1 % (w/v) osmium tetroxide vapor for 24 h, dried using LEICA EM CPD, and then sputter coated with gold using an EIKO IB-3 sputter coater. The images were taken with a HITACHI S-3400 N scanning electron microscope.

### Availability of supporting data

Raw Illumina sequences were deposited in the National Center for Biotechnology Information (NCBI) and can be accessed in the Short Read Archive (SRA) database (http://trace.ncbi.nlm.nih.gov/Traces/sra/) under accession ID SRP057292 including SRX998386 for CK and SRX1004505 for DT.

## Additional files

Additional file 1:
**Summary of the GO classifications of assembled unigenes.** (XLSX 9 kb) Additional file 2:
**Summary of the KEGG classifications of assembled unigenes.** (XLSX 10 kb) Additional file 3:
**Transcriptome CDS predicted by BLASTx and ESTScan.** (A) The length distribution of CDS using BLASTx, (B) The length distribution of proteins using BLASTx, (C) The length distribution of CDs using ESTscan, (D) The length distribution of proteins using ESTscan. (TIFF 794 kb) Additional file 4:
**The list of all functional annotation DEGs.** (XLSX 680 kb) Additional file 5:
**The heat-map cluster of DEGs. Color scale indicates fold changes of gene expression. **Increased transcript abundance is shown in red while decreased transcript abundance is shown in blue. The results show that 3134 unigenes were differentially expressed between the CK and DT samples. (PDF 126 kb) Additional file 6:
**Histogram of GO classification of the DEGs.** The results are summarized in three main GO categories: biological process, cellular component and molecular function. The x-axis indicates the subcategories, and the y-axis indicates the numbers related to the total number of GO terms present; the DEGs numbers that are assigned the same GO terms are indicated at the top of the bars. (TIFF 938 kb) Additional file 7:
**Scatterplot of the KEGG pathway enrichment of up regulated DEGs.** The x-axis indicates the Rich factor of each pathway, and the y-axis indicates the name for each pathway. Color scale indicates the q-value. The size of the spots indicates the numbers of the DEGs in each pathway. (TIFF 675 kb) Additional file 8:
**Scatterplot of the KEGG pathway enrichment of down regulated DEGs.** The x-axis indicates the Rich factor of each pathway, and the y-axis indicates the name for each pathway. Color scale indicates the q-value. The size of the spots indicates the numbers of the DEGs in each pathway. (TIFF 607 kb) Additional file 9:
**The repeat unit, length, location and primer information for EST-SSRs.** (XLSX 4088 kb) Additional file 10:
**Primer information for qRT-PCR analysis.** (XLSX 10 kb) Additional file 11:
**The figure of an agarose gel with PCR products.** (TIFF 11899 kb)

## Abbreviations

DEGs: Differentially expressed genes
PEG6000: Polyethylene glycol-6000
RWC: Relative water content
MDA: Malondialdehyde
SOD: Superoxide dismutase
POD: Peroxidase
GSH: Glutathione
NGS: Next generation sequencing
RNA-Seq: High-throughput RNA-sequencing
NCBI: National Center for Biotechnology Information
SRA: Short Read Archive
Nr: NCBI non-redundant protein sequences
Nt: NCBI non-redundant nucleotide sequences
Pfam: Protein family
GO: Gene ontology
KEGG: Kyoto Encyclopedia of Genes and Genomes
KOG/COG: Clusters of orthologous groups of database
SSRs: Simple Sequence Repeats
BP: Biological processes
MF: Molecular function
CC: Cellular component
LEA: Late embryogenesis abundant proteins
HSPs: Heat-shock proteins
CaMs: Calmodulins
CaM kinase/CDPKs: Calcium/calmodulin-dependent protein kinases
CBLs: Calcineurin B-like proteins
MAPK: Mitogen-activated protein kinase
DAG: Diacylglycerol
PLC: Phospholipase C
TFs: Transcription factors
F3H: Flavanone 3-hydroxylase
F3'5'H: Flavonoid 3’, 5’-dydroxylase
FM: Flavonoid 3’-monooxygenase
UFGT: UDP-glucose flavonoid glucosyltransferase
USP: Universal stress protein
ACCase: Acetyl-CoA carboxylase
FAS: Fatty acid synthase
ACP: Acyl carrier protein
VLCFAs: Very long chain fatty acids
FAE: Fatty acid elongase
KCS: β-ketoacyl-CoA synthase
KCR: β-ketoacyl-CoA reductase
HCD: β-hydroxyacyl-CoA dehydratase
ECR: Enoyl-CoA reductase
GPAT: Glycerol-3-phosphate acyltransferase
FAR: Fatty acyl-CoA reductase
WS/DGAT: Wax synthase/diacylglycerol acyltransferase
LACS: Long chain acyl-CoA synthetase
ER: Endoplasmic reticulum
PM: Plasma membrane
ABCG32: ATP BINDING CASSETTEG32
LTPs: Lipid transfer proteins
nsLTPs: non-specific LTPs
SEM: Scanning electron microscopy.

## References

[1] Bray EA (1997). Plant responses to water deficit. Trends Plant Sci.

[2] Lutts S, Kinet JM, Bouharmont J (1995). Changes in plant response to NaCl during development of rice (*Oryza sativa* L.) varieties differing in salinity resistance. J Exp Bot.

[3] Chaves MM, Maroco JP, Pereira JS (2003). Understanding plant responses to drought—from genes to the whole plant. Funct Plant Biol.

[4] Jenks MA, Hasegawa PM, Jain SM. Advances in molecular breeding toward drought and salt tolerant crops. Netherlands: Springer; 2007. p. 817.

[5] Valliyodan B, Nguyen HT (2006). Understanding regulatory networks and engineering for enhanced drought tolerance in plants. Curr Opin Plant Biol.

[6] Shinozaki K, Yamaguchi-Shinozaki K, Seki M (2003). Regulatory network of gene expression in the drought and cold stress responses. Curr Opin Plant Biol.

[7] Long Y, Zhang J, Tian X, Wu S, Zhang Q, Zhang J (2014). De novo assembly of the desert tree *Haloxylon ammodendron* (*C. A. Mey*.) based on RNA-Seq data provides insight into drought response, gene discovery and marker identification. BMC Genomics.

[8] Zhou Y, Fei G, Ran L, Feng J, Li H (2012). De novo sequencing and analysis of root transcriptome using 454 pyrosequencing to discover putative genes associated with drought tolerance in *Ammopiptanthus mongolicus*. BMC Genomics.

[9] Liu Y, Liu M, Li X, Cao B, Ma X (2014). Identification of differentially expressed genes in leaf of *Reaumuria soongorica* under PEG-induced drought stress by digital gene expression profiling. Plos One.

[10] Lu H, Wang SS, Zhou QW, Zhao YN, Zhao BY (2013). Damage and control of major poisonous plants in the western grasslands of China–a review. Rangeland J.

[11] Zhang Y, Zhao D, Li Y (2007). The research progress of *Cynanchum komarovii*. J Agric Sci.

[12] Mi H, Xu X, Li S, He J, Wei Y, Li Q (2003). Effects of drought stress on system of defense enzymes and RWC and membrane electrolyte in *Cynanchum komarovii* seedlings. Acta Bot Boreali-Occidential Sinica.

[13] Mi H, Xu X, Li S, He J, Zhang Y, Zhao T (2004). Effects of soil water stress on contents of chlorophyll, soluble sugar, starch, C/N of two desert plants (*Cynanchum komarovii* and *Glycyrrhiza uralensis*). Acta Bot Boreali-Occidential Sinica.

[14] Yang L, Yu C, Shi F, Wei Y, Wang C, Hu H (2007). Effects of abscisic acid on growth and dehydration tolerance of *Cynanchum komarovii* seedlings. Plant Growth Regul.

[15] Chen C, Zhao X, Li X (2012). Osmotic adjustment mechanism of *Cynanchum komarovii* under drought stress. J Desert Res.

[16] Varshney RK, Nayak SN, May GD, Jackson SA (2009). Next-generation sequencing technologies and their implications for crop genetics and breeding. Trends Biotechnol.

[17] Li C, Deng G, Yang J, Viljoen A, Jin Y, Kuang R (2012). Transcriptome profiling of resistant and susceptible Cavendish banana roots following inoculation with *Fusarium oxysporum* f. sp. cubense tropical race 4. BMC Genomics.

[18] Bhardwaj J, Chauhan R, Swarnkar MK, Chahota RK, Singh AK, Shankar R (2013). Comprehensive transcriptomic study on horse gram (*Macrotyloma uniflorum*): De novo assembly, functional characterization and comparative analysis in relation to drought stress. BMC Genomics.

[19] Wu Y, Wei W, Pang X, Wang X, Zhang H, Dong B (2014). Comparative transcriptome profiling of a desert evergreen shrub, *Ammopiptanthus mongolicus*, in response to drought and cold stresses. BMC Genomics.

[20] Dang ZH (2013). Transcriptomic profiling of the salt-stress response in the wild recretohalophyte *Reaumuria trigyna*. BMC Genomics.

[21] Scott KD, Eggler P, Seaton G, Rossetto M, Ablett EM, Lee LS (2000). Analysis of SSRs derived from grape ESTs. Theor Appl Genet.

[22] Zhu J (2002). Salt and drought stress signal transduction in plants. Annu Rev Plant Biol.

[23] Bowler C, Fluhr R (2000). The role of calcium and activated oxygens as signals for controlling cross-tolerance. Trends Plant Sci.

[24] Knight H (1999). Calcium signaling during abiotic stress in plants. Int Rev Cytol vol.

[25] Ranty B, Aldon D, Galaud J-P (2006). Plant calmodulins and calmodulin-related proteins: multifaceted relays to decode calcium signals. Plant Signal Behav.

[26] Cristina MS, Petersen M, Mundy J (2010). Mitogen-activated protein kinase signaling in plants. Annu Rev Plant Biol.

[27] Xu J, Zhang S (2015). Mitogen-activated protein kinase cascades in signaling plant growth and development. Trends Plant Sci.

[28] Estavillo GM, Crisp PA, Pornsiriwong W, Wirtz M, Collinge D, Carrie C (2011). Evidence for a SAL1-PAP chloroplast retrograde pathway that functions in drought and high light signaling in *Arabidopsis*. Plant Cell.

[29] Xiong L, Schumaker KS, Zhu J-K. Cell signaling during cold, drought, and salt stress. Plant Cell. 2002;14(suppl1):S165-S183. 10.1105/tpc.000596 PMC151254 12045276

[30] Shinozaki K, Yamaguchi-Shinozaki K (2007). Gene networks involved in drought stress response and tolerance. J Exp Bot.

[31] Singh K, Foley R, OñateSánchez L (2002). Transcription factors in plant defense and stress responses. Curr Opin Plant Biol.

[32] Wang W, Vinocur B, Shoseyov O, Altman A (2004). Role of plant heat-shock proteins and molecular chaperones in the abiotic stress response. Trends Plant Sci.

[33] Winkel-Shirley B (2002). Biosynthesis of flavonoids and effects of stress. Curr Opin Plant Biol.

[34] Wang M, Li P, Li C, Pan Y, Jiang X, Zhu D (2014). SiLEA14, a novel atypical LEA protein, confers abiotic stress resistance in foxtail millet. BMC Plant Biol.

[35] Kuromori T, Miyaji T, Yabuuchi H, Shimizu H, Sugimoto E, Kamiya A (2010). ABC transporter AtABCG25 is involved in abscisic acid transport and responses. Proc Natl Acad Sci.

[36] Wang M, Xu Q, Yu J, Yuan M (2010). The putative *Arabidopsis* zinc transporter ZTP29 is involved in the response to salt stress. Plant Mol Biol.

[37] Guo F, Young J, Crawford NM (2003). The nitrate transporter AtNRT1. 1 (CHL1) functions in stomatal opening and contributes to drought susceptibility in *Arabidopsis*. Plant Cell.

[38] Alexandersson E, Fraysse L, Sjövall-Larsen S, Gustavsson S, Fellert M, Karlsson M (2005). Whole gene family expression and drought stress regulation of aquaporins. Plant Mol Biol.

[39] Reddy AR, Chaitanya KV, Vivekanandan M (2004). Drought-induced responses of photosynthesis and antioxidant metabolism in higher plants. J Plant Physiol.

[40] Pollard M, Beisson F, Li Y, Ohlrogge JB (2008). Building lipid barriers: biosynthesis of cutin and suberin. Trends Plant Sci.

[41] Shepherd T, Wynne Griffiths D (2006). The effects of stress on plant cuticular waxes. New Phytol.

[42] Kosma DK, Bourdenx B, Bernard A, Parsons EP, Lü S, Joubès J (2009). The impact of water deficiency on leaf cuticle lipids of *Arabidopsis*. Plant Physiol.

[43] Borisjuk N, Hrmova M, Lopato S (2014). Transcriptional regulation of cuticle biosynthesis. Biotechnol Adv.

[44] Lee S, Suh M (2013). Recent advances in cuticular wax biosynthesis and its regulation in *Arabidopsis*. Mol Plant.

[45] Bernard A, Domergue F, Pascal S, Jetter R, Renne C, Faure J-D (2012). Reconstitution of plant alkane biosynthesis in yeast demonstrates that *Arabidopsis* ECERIFERUM1 and ECERIFERUM3 are core components of a very-long-chain alkane synthesis complex. Plant Cell.

[46] Jessen D, Olbrich A, Knüfer J, Krüger A, Hoppert M, Polle A (2011). Combined activity of LACS1 and LACS4 is required for proper pollen coat formation in *Arabidopsis*. Plant J.

[47] Höfer R, Briesen I, Beck M, Pinot F, Schreiber L, Franke R (2008). The *Arabidopsis* cytochrome P450 CYP86A1 encodes a fatty acid ω-hydroxylase involved in suberin monomer biosynthesis. J Exp Bot.

[48] Xiao F, Mark Goodwin S, Xiao Y, Sun Z, Baker D, Tang X (2004). *Arabidopsis CYP86A2* represses *Pseudomonas syringae* type III genes and is required for cuticle development. EMBO J.

[49] Yang W, Pollard M, Li-Beisson Y, Beisson F, Feig M, Ohlrogge J (2010). A distinct type of glycerol-3-phosphate acyltransferase with sn-2 preference and phosphatase activity producing 2-monoacylglycerol. Proc Natl Acad Sci.

[50] Girard A, Mounet F, Lemaire-Chamley M, Gaillard C, Elmorjani K, Vivancos J (2012). Tomato GDSL1 is required for cutin deposition in the fruit cuticle. Plant Cell.

[51] Bessire M, Borel S, Fabre G, Carraça L, Efremova N, Yephremov A (2011). A member of the PLEIOTROPIC DRUG RESISTANCE family of ATP binding cassette transporters is required for the formation of a functional cuticle in *Arabidopsis*. Plant Cell.

[52] Bernard A, Joubès J (2013). *Arabidopsis* cuticular waxes: advances in synthesis, export and regulation. Prog Lipid Res.

[53] Aharoni A, Dixit S, Jetter R, Thoenes E, van Arkel G, Pereira A (2004). The SHINE clade of AP2 domain transcription factors activates wax biosynthesis, alters cuticle properties, and confers drought tolerance when overexpressed in *Arabidopsis*. Plant Cell.

[54] Grabherr MG, Haas BJ, Yassour M, Levin JZ, Thompson DA, Amit I (2011). Full-length transcriptome assembly from RNA-Seq data without a reference genome. Nat Biotechnol.

[55] Conesa A, Götz S, García-Gómez JM, Terol J, Talón M, Robles M (2005). Blast2GO: a universal tool for annotation, visualization and analysis in functional genomics research. Bioinformatics.

[56] Wang L, Feng Z, Wang X, Wang X, Zhang X (2010). DEGseq: an R package for identifying differentially expressed genes from RNA-seq data. Bioinformatics.

[57] Benjamini Y, Hochberg Y. Controlling the false discovery rate: a practical and powerful approach to multiple testing. J Roy Stat Soc Ser B (Methodological). 1995;289–300.

[58] Young MD, Wakefield MJ, Smyth GK, Oshlack A (2010). Gene ontology analysis for RNA-seq: accounting for selection bias. Genome Biol.

[59] Wallenius KT. Biased sampling: the non-central hypegeometric probability distribution. PhD diss. Stanford University 1963.

[60] Mao X, Cai T, Olyarchuk JG, Wei L (2005). Automated genome annotation and pathway identification using the KEGG Orthology (KO) as a controlled vocabulary. Bioinformatics.

[61] Wang Q, Li F, Zhang X, Zhang Y, Hou Y, Zhang S (2011). Purification and characterization of a CkTLP protein from *Cynanchum komarovii* seeds that confers antifungal activity. Plos One.

