# Supplementary figures and images for: De novo transcriptome sequencing and comprehensive analysis of the drought-responsive genes in the desert plant Cynanchum komarovii
Source: BMC Genomics. 2015 Oct 6;16:753. doi: 10.1186/s12864-015-1873-x (PMC4594960; doi:10.1186/s12864-015-1873-x)

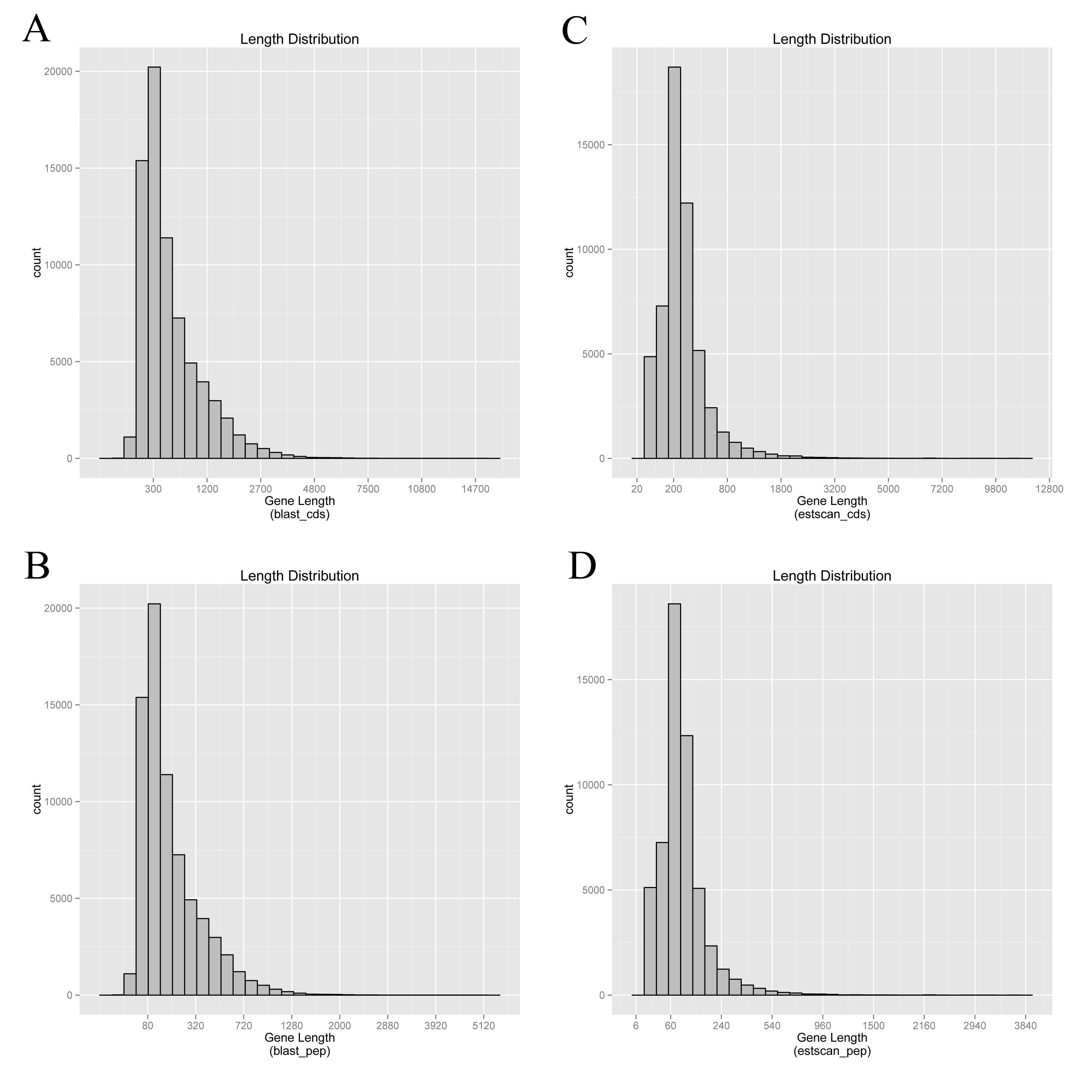

Supplement: Additional file 3: — Transcriptome CDS predicted by BLASTx and ESTScan. (A) The length distribution of CDS using BLASTx, (B) The length distribution of proteins using BLASTx, (C) The length distribution of CDs using ESTscan, (D) The length distribution of proteins using ESTscan. (TIFF 794 kb) [file 12864_2015_1873_MOESM3_ESM.tif]

Cluster analysis of differentially expressed genes

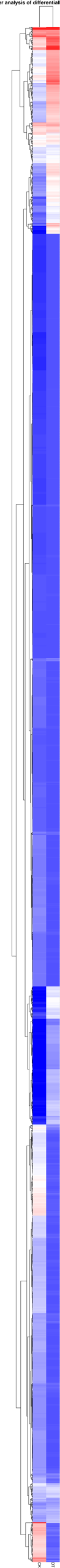

Supplement: Additional file 5: — The heat-map cluster of DEGs. Color scale indicates fold changes of gene expression. Increased transcript abundance is shown in red while decreased transcript abundance is shown in blue. The results show that 3134 unigenes were differentially expressed between the CK and DT samples. (PDF 126 kb) [file 12864_2015_1873_MOESM5_ESM.pdf]

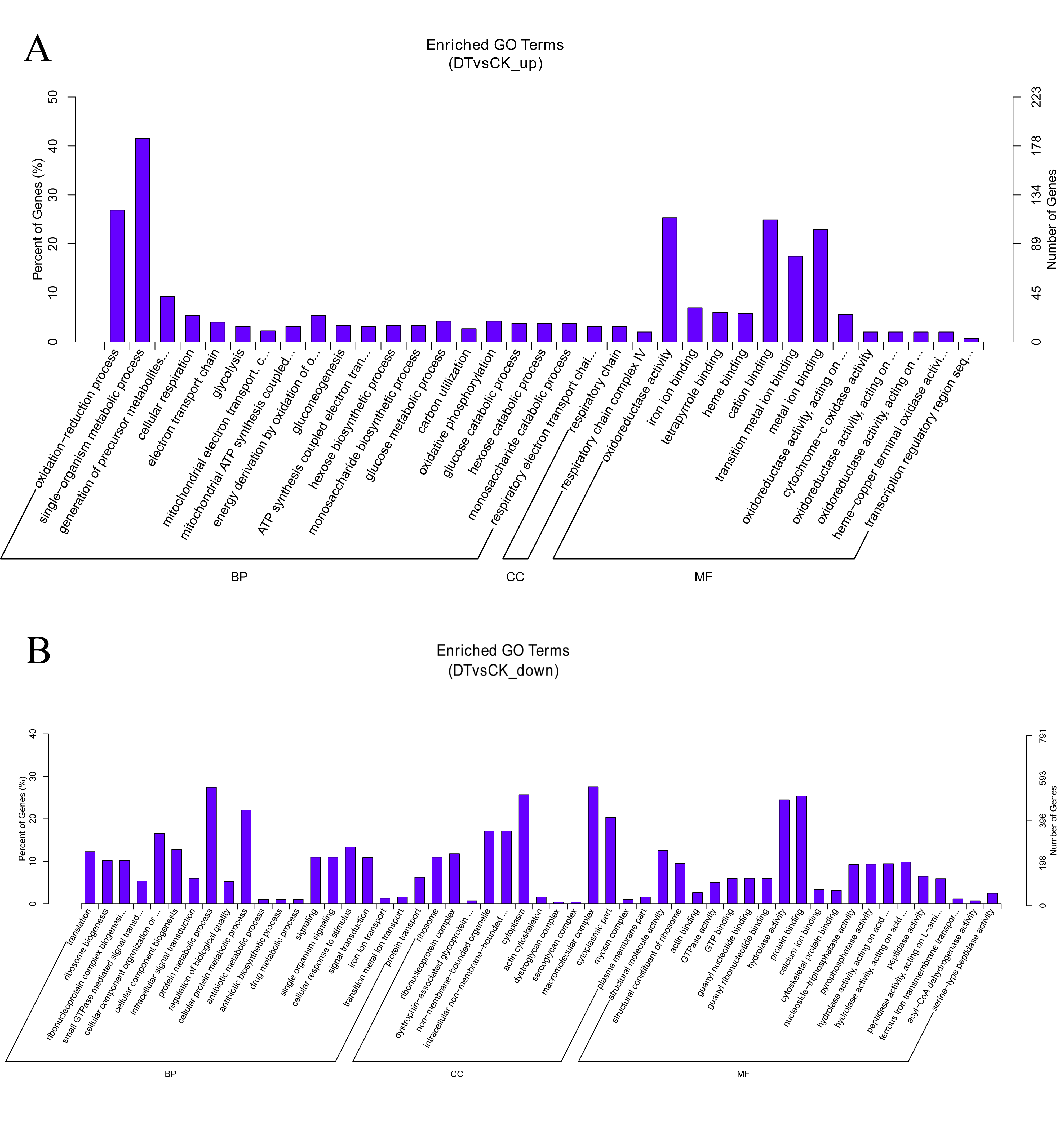

Supplement: Additional file 6: — Histogram of GO classification of the DEGs. The results are summarized in three main GO categories: biological process, cellular component and molecular function. The x-axis indicates the subcategories, and the y-axis indicates the numbers related to the total number of GO terms present; the DEGs numbers that are assigned the same GO terms are indicated at the top of the bars. (TIFF 938 kb) [file 12864_2015_1873_MOESM6_ESM.tif]

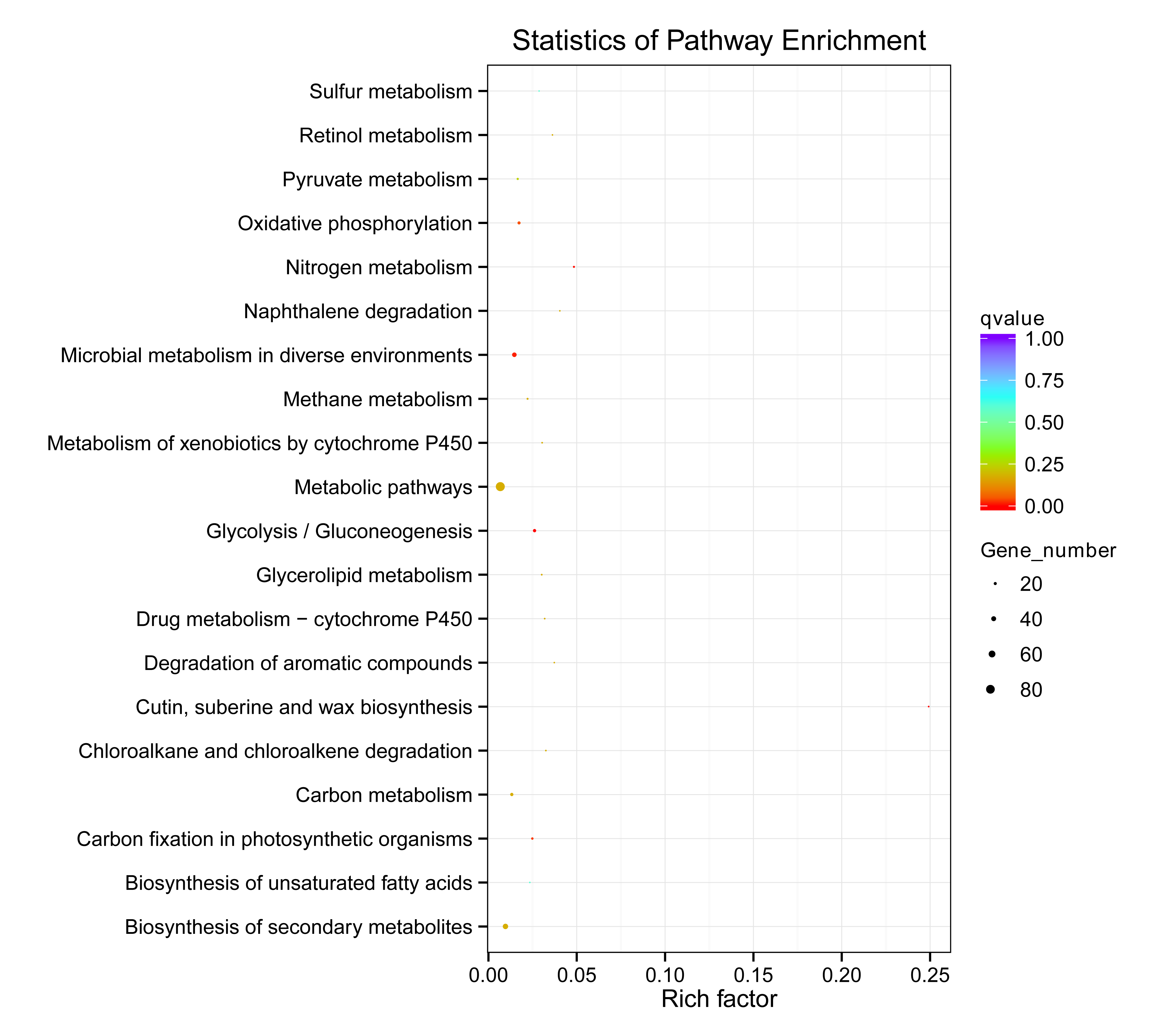

Supplement: Additional file 7: — Scatterplot of the KEGG pathway enrichment of up regulated DEGs. The x-axis indicates the Rich factor of each pathway, and the y-axis indicates the name for each pathway. Color scale indicates the q-value. The size of the spots indicates the numbers of the DEGs in each pathway. (TIFF 675 kb) [file 12864_2015_1873_MOESM7_ESM.tif]

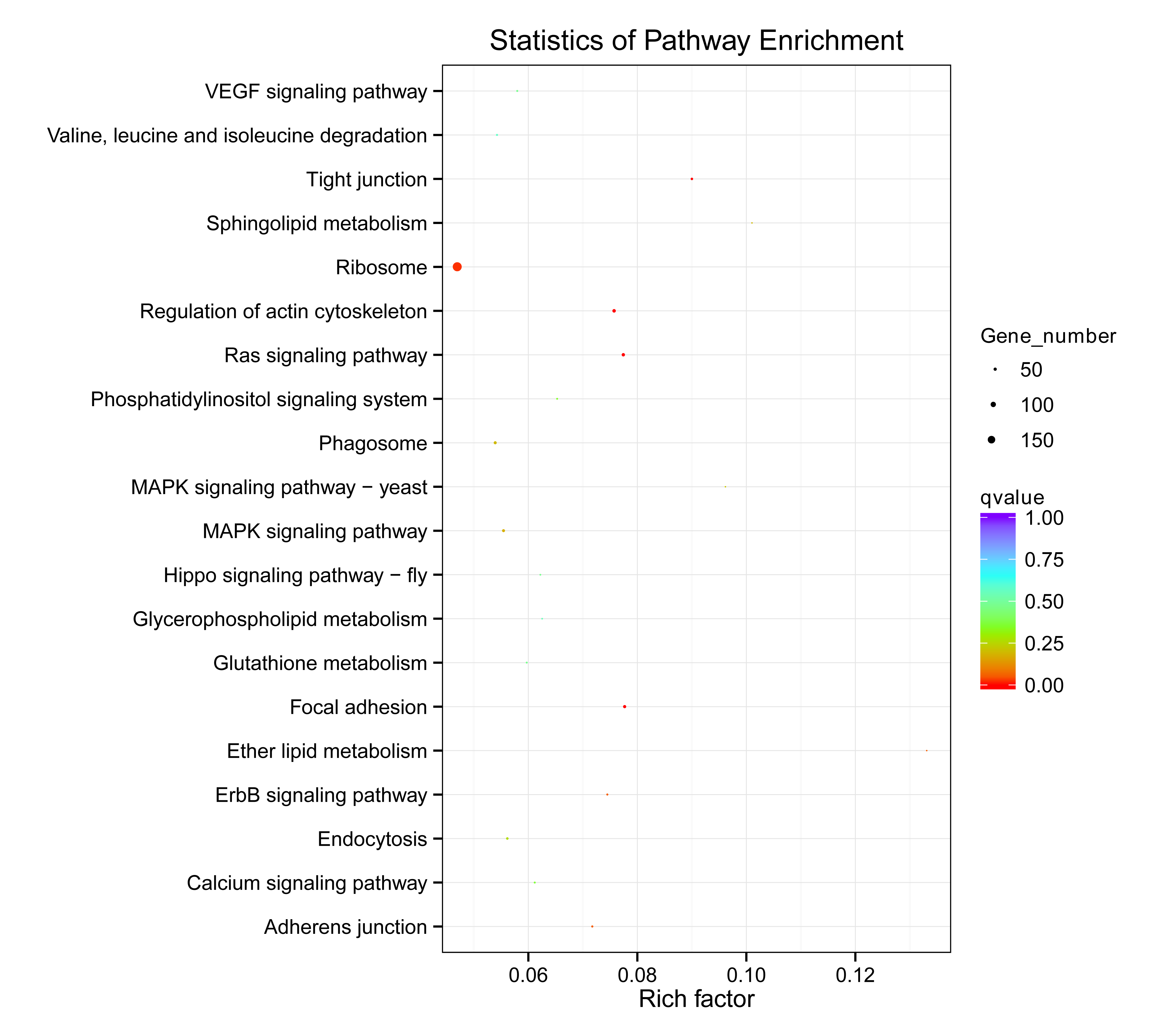

Supplement: Additional file 8: — Scatterplot of the KEGG pathway enrichment of down regulated DEGs. The x-axis indicates the Rich factor of each pathway, and the y-axis indicates the name for each pathway. Color scale indicates the q-value. The size of the spots indicates the numbers of the DEGs in each pathway. (TIFF 607 kb) [file 12864_2015_1873_MOESM8_ESM.tif]

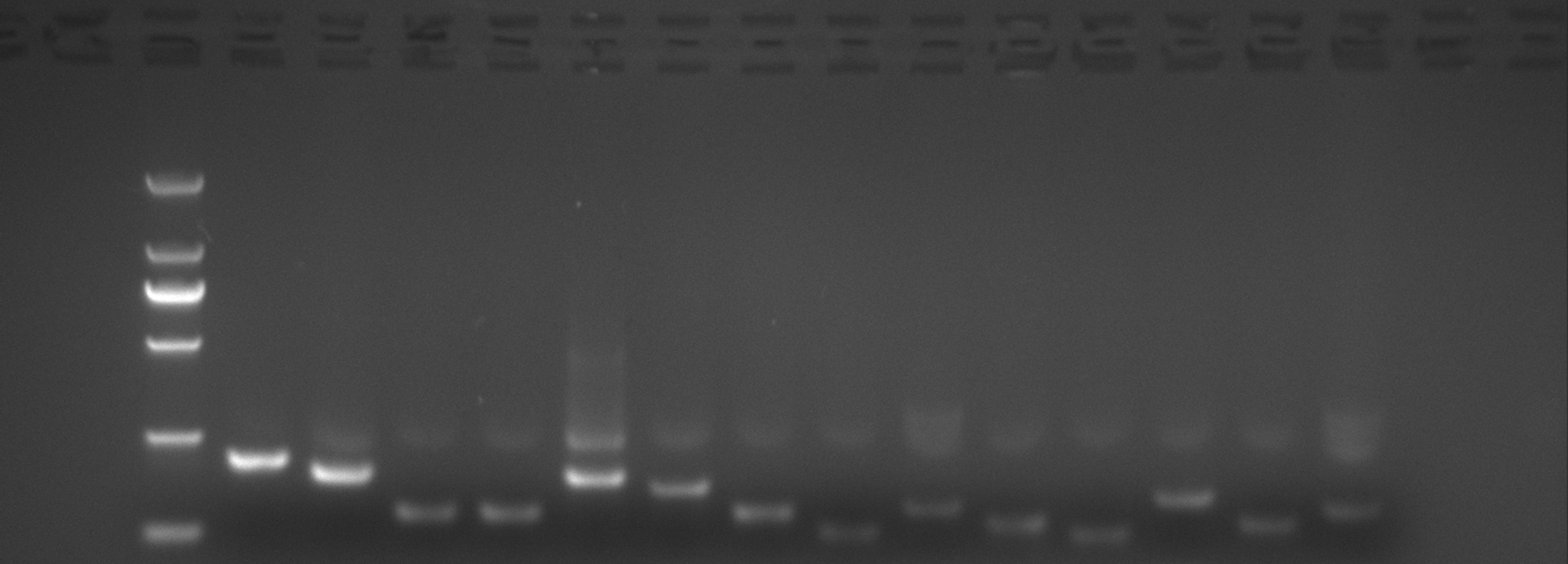

Supplement: Additional file 11: — The figure of an agarose gel with PCR products. (TIFF 11899 kb) [file 12864_2015_1873_MOESM11_ESM.tif]
